# Introduction of benzyloxy pharmacophore into aryl/heteroaryl chalcone motifs as a new class of monoamine oxidase B inhibitors

**DOI:** 10.1038/s41598-022-26929-x

**Published:** 2022-12-27

**Authors:** Sachithra Thazhathuveedu Sudevan, Jong Min Oh, Mohamed A. Abdelgawad, Mohammed A. S. Abourehab, T. M. Rangarajan, Sunil Kumar, Iqrar Ahmad, Harun Patel, Hoon Kim, Bijo Mathew

**Affiliations:** 1 Department of Pharmaceutical Chemistry, Amrita School of Pharmacy, Amrita Vishwa Vidyapeetham, AIMS Health Sciences Campus, Kochi, 682 041 India; 2 Department of Pharmacy, and Research Institute of Life Pharmaceutical Sciences, Sunchon National University, Suncheon, 57922 Republic of Korea; 3 Department of Pharmaceutical Chemistry, College of Pharmacy, Jouf University, Sakaka, 72341 Saudi Arabia; 4 Pharmaceutical Organic Chemistry Department, Faculty of Pharmacy, Beni-Suef University, Beni-Suef, 62514 Egypt; 5 Department of Pharmaceutics, College of Pharmacy, Umm Al-Qura University, Makkah, 21955 Saudi Arabia; 6 Department of Chemistry, Sri Venketeswara College, University of Delhi, New Delhi, 110021 India; 7 Department of Pharmaceutical Chemistry, Prof. Ravindra Nikam College of Pharmacy, Gondur, Dhule, 424002 Maharashtra India; 8 Division of Computer Aided Drug Design, Department of Pharmaceutical Chemistry, R. C. Patel Institute of Pharmaceutical Education and Research, Shirpur, 425405 Maharashtra India

**Keywords:** Biochemistry, Biological techniques, Drug discovery

## Abstract

The inhibitory action of fifteen benzyloxy *ortho*/*para*-substituted chalcones (**B1-B15**) was evaluated against human monoamine oxidases (hMAOs). All the molecules inhibited hMAO-B isoform more potently than hMAO-A. Furthermore, the majority of the molecules showed strong inhibitory actions against hMAO-B at 10 μM level with residual activities of less than 50%. Compound **B10** has an IC_50_ value of 0.067 μM, making it the most potent inhibitor of hMAO-B, trailed by compound **B15** (IC_50_ = 0.12 μM). The thiophene substituent (**B10**) in the **A**-ring exhibited the strongest hMAO-B inhibition structurally, however, increased residue synthesis did not result in a rise in hMAO-B inhibition. In contrast, the benzyl group at the *para* position of the **B**-ring displayed more hMAO-B inhibition than the other positions. Compounds **B10** and **B15** had relatively high selectivity index (SI) values for hMAO-B (504.791 and 287.600, respectively). K_i_ values of **B10** and **B15** were 0.030 ± 0.001 and 0.033 ± 0.001 μM, respectively. The reversibility study showed that **B10** and **B15** were reversible inhibitors of hMAO-B. PAMPA assay manifested that the benzyloxy chalcones (**B10** and **B15**) had a significant permeability and CNS bioavailability with *Pe* value higher than 4.0 × 10^–6^ cm/s. Both compounds were stabilized in protein–ligand complexes by the π-π stacking, which enabled them to bind to the hMAO-B enzyme's active site incredibly effectively. The hMAO-B was stabilized by **B10**- and **B15**-hMAO-B complexes, with binding energies of − 74.57 and − 87.72 kcal/mol, respectively. Using a genetic algorithm and multiple linear regression, the QSAR model was created. Based on the best 2D and 3D descriptor-based QSAR model, the following statistics were displayed: R^2^ = 0.9125, Q^2^_loo_ = 0.8347. These findings imply that **B10** and **B15** are effective, selective, and reversible hMAO-B inhibitors.

## Introduction

Following Alzheimer's disease (AD), Parkinson's disease (PD) is by far the second most prevalent neurological condition. Persistent atrophy of dopaminergic neurons in the substantia nigra (SN) pars compacta is indeed a pathological attribute of PD. Non-dopaminergic systems, including noradrenergic, serotonergic, as well as cholinergic ones, are nevertheless, apparently implicated in its path mechanisms^1–3^. Contemporary therapy strategies endeavor to replenish dopaminergic deficits perhaps by boosting dopamine directly, suppressing dopamine disintegration, or functioning as an agonist on dopaminergic sites. Nevertheless, there seems to be currently no neuroprotective medication adequate for arresting the onset of PD^4–7^. Levodopa affords the greatest clinical impact and thus has been the benchmark of therapy ever since its debut in the 1960s. Dopamine agonists (DAGs), catechol-*O* methyltransferase (COMT) inhibitors, as well as monoamine oxidase (MAO)-B inhibitors, possess entrenched spots in the management of PD in addition to levodopa^8–11^. MAO has two isoforms: MAO-A and MAO-B.

In the gastrointestinal system, MAO-A is the major isoform; whereas, in the human brain, the MAO-B isoform prevails, degrading dopamine into 3,4-dihydroxyphenylacetic acid as well as homovanillic acid^12^. Mitochondrial disruption, as well as oxidative stress, is triggered by unstable dopamine residues and appears crucially in the pathogenic phase of PD. MAO-B biochemically transforms both endogenous and exogenous dopamine into hydrogen peroxide, rendering it imperative for its underlying processes in PD oxidative stress and oxidative deterioration. Raised MAO-B levels have been implicated in aging and specific neurological ailments like AD and PD, showing a pathology presumed to be attributable to the enhanced oxidative stress, which ensues in such circumstances^13^. 1-Methyl-4-phenylpyridinium ion (MPP^+^) triggers experimental or secondary parkinsonism, while MAO-B enables its transformation from 1-methyl-4-phenyl1,2,3,6-tetrahydropyridine (MPTP)^14^. Both irreversible and reversible MAO inhibitors have been studied. As non-selective monoamine oxidase (MAO) inhibitors, the USFDA approved phenelzine, isocarboxazid, and tranylcypromine. There remained a necessity for reversible inhibitors, since the preponderance of the pioneer MAO inhibitors constituted irreversible type. Subsequently, the least lethal and greater efficacious selective MAO-A (like moclobemide) inhibitors, as well as MAO-B (like selegiline) inhibitors, had been devised^15^. Owing to the medicinal potential of MAO inhibitors across several neurodegenerative conditions, these MAO inhibitors had been actively developed^16,17^. Three MAO-B inhibitors such as selegiline, rasagiline, and safinamide, were being used often to manage PD.

The selective MAO-B inhibitor safinamide was approved to manage PD in 2017. Safinamide, the first antiparkinson drug to be authorized in the decade, also prevents the reuptake of dopamine and serotonin as well as glutamate release. Although it has a reversible effect, unlike the preceding antiparkinson drugs selegiline and rasagiline, it also has a positive pharmacokinetic pattern and a bioavailability of about 95%^18^. Safinamide travels via a complicated metabolic pathway that ends with inactive metabolites^19^. Safinamide inhibits levodopa-derived dopamine from being rendered inert either endogenously or exogenously, thereby raising the brain's dopamine tolerance. Safinamide has a favorable attitude for MAO-B in comparison to selegiline and rasagiline, therefore, at therapeutic concentrations, there is no MAO-A inhibition. Safinamide's inhibition of MAO-B is transient, owing to its non-covalent allegiance to MAO-B, thus reducing drug contention^20^.

The adoption of safinamide as an alternative to levodopa in individuals with extreme Parkinson's illness demonstrated considerable advances in physical function. Fewer levodopa dosages have been used due to safinamide. With such a low failure rate, safinamide was well tolerated^21^. In drug testing, there was no noticeable difference between safinamide and a placebo in the incidence of adverse effects like nausea, dizziness, exhaustion, sleeplessness, and headache. With such a pharmacokinetic profile that enables once-daily administration, rasagiline and safinamide were shown to enhance congruence in PD patients. Despite not displaying obvious deleterious impacts, safinamide had been revealed to be inadequate to carry out its therapeutic purpose in late-stage human clinical studies. As a corollary, the design and replication of the core feature of this scaffolding culminate in improved medical innovation^22^.

**Figure 1:**
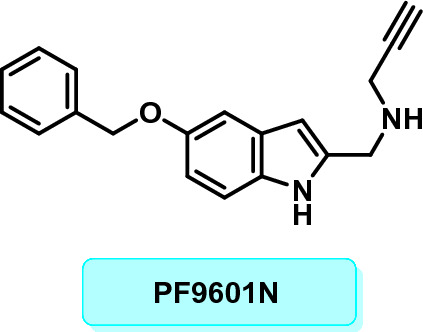
PF9601N.

Safinamide has a unique architecture, owing to the apparent benzyloxy pharmacophore on the phenyl ring and also the aminoamide on the relevant *para* positioning. Even though the chemical context, in which each of all these entities operates regarding MAO-B functioning, is quite distinct, their exclusivity can also be applied to a broad array of swapped as well as spherical moieties having controllable suppressive implications^23^. The existing research reported that the presence of the benzyloxy group in safinamide^20,22^, indolalkylamines^24,25^; where the discovery as well as investigation of N-((5-(benzyloxy)-1H-indol-2-yl)methyl)prop-2-yn-1-amine ("PF9601N") provided the very initial observation and reported the implications of a "benzyloxy" motif linked to an aryl/heteroaryl ring. In accordance with the study, the acetylenic or allenic moiety's natures have minimal bearing on the degree of selectivity or potency, despite the fact that neither of the non-acetylenic or allenic 2-indolylmethylamines are selective inhibitors; however, it appears to be an essential requirement for selectivity (Fig. 1)^26,27^, caffeines^28,29^, oxadiazolone^30–33^, indoles, β-nitrostyrenes^34^, coumarins^35–39^, benzoquinones^40^, α-tetralones^41^, benzofurans^42^, chromones and chromanone analogues^43–45^ has made them effective MAO inhibitors^30–33,46^.

Chalcone is an α,β-unsaturated ketone, which contributes to the brief spacing in the straightforward chemical structure^47^. The olefinic attachment in chalcones enables the generation of both *cis* and *trans* isomers, however, the thermodynamically highly persistent *trans* form is much more prevalent than the *cis* form. Additionally, this unique conjugated kind of ketone performs as a Michael acceptor in numerous physiological signal transduction cascades in cells^48^. The electrophilic propensity of the enone's entity is driven by the charge distribution discrepancy of the aromatic core induced by the inclusion of diverse substituents^49^. These molecules are shown to possess a broad variety of physiological functions, such as the aptitude to suppress human MAOs (hMAOs). One of the two hMAO isotypes can be inhibited potently as well as preferentially by amending the scaffolding and meticulously considering the substituents inserted on the **A** and/or **B** rings. Relying on the linkers appended to the **A**/**B** rings, the conventional chalcones having these two aromatic rings as heads are inhibitors of hMAO-B in a low molar range. When compounds like furan and thiophene are incorporated into the **A** ring, such molecules often benefit from electron-donating lipophilic units coupled to the **B** ring. The halogens are frequently extensively endured, to varying degrees, and might improve the lipophilicity aiding in encounters in the entry chamber. The nitro and hydroxyl groups on **B** ring often contribute to a decline in interaction towards hMAO-B and the congestion of hydroxy or methoxy units on the corresponding ring. The inclusion of the hydroxyl group on ring **A**, on either hand, is effectively sustained, notably if halogens were bound to **B** ring. This molecule might create hydrogen bonds with tyrosine hydroxyl groups or water molecules within the enzyme cavities, strengthening the enzyme interactions^50–53^.

In light of these observations, the current work outlines the synthesis of a series of chalcones coupled to benzyloxy moieties and explores the compound’s in vitro hMAO-A as well as hMAO-B inhibitory characteristics. The principal compounds were also put through extensive testing in the areas of kinetics, reversibility, blood–brain barrier (BBB) permeation, and molecular dynamics (MD) simulation.

## Materials and methods

### Synthesis

**Scheme 1:**
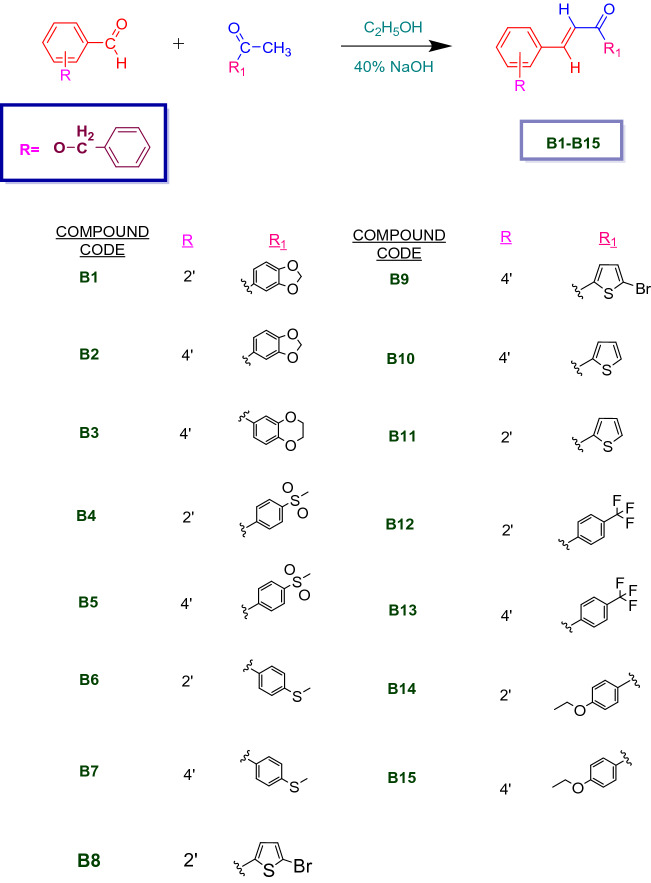
Synthesis of benzyloxy chalcones (**B1**–**B15**).

An equimolar mixture of 0.01 M of various substituted acetophenones, para/ortho-substituted benzyloxy benzaldehyde, 25 mL ethanol, and 7.5 mL 40% KOH was stirred for 20 to 24 h to synthesize the benzyloxy chalcones. Upon pouring the obtained mixture to crushed ice, the yellow solid product was obtained as precipitates which was then filtered by suction. It was dried after being washed with water. Employing methanol or ethanol, the dried products were recrystallized (Scheme 1). To monitor all of the reactions, thin layer chromatography was carried out on pre-coated TLC plates (Silica gel 60-120#) using the solvent system hexane:ethyl acetate (9:1).

### Inhibition studies of hMAO-A and hMAO-B

Recombinant hMAO-A and hMAO-B were used in the hMAO inhibitory activity assay with substrates kynuramine and benzylamine of 0.06 mM and 0.3 mM concentrations, respectively^54,55^. It was determined that the benzylamine-MAO-B Km ranged from 0.25 to 0.33 mM for kinetics. Toloxatone and clorgyline were served as reference hMAO-A inhibitors, while pargyline and Lazabemide had been used as reference hMAO-B inhibitors. From Sigma-Aldrich (St. Louis, MO, USA), enzymes, substrates, and reference chemicals were purchased.

### Enzyme inhibition and kinetic studies

GraphPad Prism software 5 was employed to assess the activity at various doses of the compounds and to calculate the IC_50_ value for the compound exhibiting a residual activity of less than 50%^56,57^. The inhibition effect was initially tested at a concentration of 10 μM. IC_50_ of hMAO-A/IC_50_ of hMAO-B was used to obtain the SI value of hMAO-B^58^. The enzyme kinetics of compounds **B10** and **B15** were assessed using hMAO-B at five different substrate dosages (0.0375–0.6 μM)^59^. The Lineweaver–Burk plots as well as their secondary plots were compared in order to investigate and ascertain the kinetic patterns.

### Reversibility analysis of B10 and B15

After preincubation for 30 min at ~ 2 × IC_50_, as previously described, the reversibility of **B10** and **B15**'s hMAO-B inhibitions was assessed^60^. The reference for reversible hMAO-B inhibitor, lazabemide, and the reference for irreversible hMAO-B inhibitor, pargyline, were preincubated at ~ 2 × IC_50_ (0.22 and 0.28 μM, respectively) for comparing with lead molecules. By contrasting the activities of dialyzed (A_D_) and undialyzed (A_U_) samples, reversibility patterns were investigated and determined.

### Blood–brain barrier (BBB) permeability study

Initial drug research utilized the parallel artificial membrane permeation assay (PAMPA) method to forecast drugs' passive, transcellular permeation via the BBB. A "sandwich" structure was developed in PAMPA using a microtiter plate with 96 wells and a Millipore filter plate with 96 wells (ipvh, 125 μm thick filter, 0.45 μm pore), which was then drenched in 0.1 mL of n-dodecane. Drug sample stock solutions in DMSO were made at 10 mM concentration and retained at 0 °C until utilization. To accomplish a final sample concentration (0.01, 0.1, and 1 mM) and limit the DMSO concentration to 1% (v/v), the stock solution was diluted in buffer at pH 7.4, prior being incorporated into a 96-well filter plate. The donor wells received 270 μL of the final dilutions, while the acceptor well received 200 μL of pH 7.4 buffer. To create a sandwich, the acceptor filter plate was properly positioned atop of the donor plate (comprising of a synthetic lipid membrane in the center, an aqueous receiver on atop, and an aqueous donor carrying an analyte on the bottom). Accessing the acceptor well from across the lipid membrane, the test substance diffuses from the donor well. The penetration supposedly happened with the sandwich intact. The UV spectrometry was employed to quantify the drug concentration in the reference, donor, and receiver wells. The following expression was employed to figure out the rate of penetration^61^.

$${\text{Log}}\;Pe = - \ln \;\left[ {1 - {\text{C}}_{{\text{A}}} /{\text{E}}_{{{\text{quilibrium}}}} } \right]/{\text{A}} \times \left( {1/{\text{V}}_{{\text{D}}} + \, 1/{\text{V}}_{{\text{A}}} } \right) \times {\text{t}}$$

where, *Pe* is permeability (cm/s), C_A_ = receptor concentration, A = area of effective filtration (0.3 cm^2^), V_D_ = donor volume (mL), V_A_ = acceptor volume (mL), t = time of incubation (s) and

$${\text{E}}_{quilibrium} = \left( {{\text{C}}_{{\text{D}}} \times {\text{V}}_{{\text{D}}} + {\text{ C}}_{{\text{A}}} \times {\text{V}}_{{\text{A}}} } \right)/\left( {{\text{V}}_{{\text{D}}} + {\text{V}}_{{\text{A}}} } \right)$$

### Molecular docking

The crystallographic structure of hMAO-B protein with accession code 2V5Z was downloaded from the PDB database RCSB. The *Protein Preparation Wizard* incorporated into Maestro preprocessed the protein's crystal 3D structure by fixing bond ordering, removing unwanted components, and fixing problems with the protein's structure like missing atoms, loops, or side chains^62,63^. The centroid box was identified using the grid generation technique based on the co-crystallized ligand to define the binding site. The 2D structure of the synthesized **B10** and **B15** compounds, and low energy 3D conformers with appropriate bond lengths and angles were obtained. At a physiological pH of 7.2 ± 0.2, the potential ionization states for each ligand structure were generated. The Glide module of the Schrödinger module was used to accomplish the docking, while all other parameters were left at their default settings^64,65^.

### Molecular dynamic (MD) simulation

MD studies were performed for the docking poses of synthesized compounds **B10** and **B15** with the lowest negative scores, i.e., top-docking poses, with an NVIDIA Quadro 6000 graphics processing unit, using the Desmond MD simulation program. More information for MD investigations (box type, thermostat and barometer settings, short- and long-range interactions calculations, etc.) can be found in previous studies because the same settings were used for the examined systems here., 100 ns of MD production was performed, where coordinates were saved at 100 ps to generate trajectories of 10,000 frames each for investigation of protein–ligand interaction dynamics^66–69^. The mean relative binding free energies were calculated using Molecular Mechanics Generalized Born Surface Area (MM/GBSA) with the thermal_mmgbsa.py script from the Prime module available in the Schrödinger suite. Principle component analysis (PCA) was executed with the trj_essential.py script to investigate protein–ligand confirmations and significant global movements upon ligand binding.

### 2D and 3D QSAR modeling

#### QSARINS-based MLR model

ChemBioDraw V.14.0 was used to draw all of the structures of the benzyloxy chalcones. Additionally, the Chem3D tool was used to transform these 2D structures into 3D shapes. Lastly, molecular descriptor (2D and 3D) computations were performed using PaDEL and RDKit. 2D and 3D MRL model were created by QSARINS version 2.2.2^70–72^.

## Results and discussion

### Chemistry

The benzyloxy chalcones were synthesized by the overnight stirring of various substituted acetophenones and *para/ortho*-substituted benzyloxy benzaldehyde via the Claisen-Schimdt reaction (Scheme 1)^73–76^. All final derivatives were characterized using ^1^H NMR, ^13^C NMR, and mass spectrometry (Supporting information Figs. S1–S33). For compounds **B10** and **B15**, the ^1^H NMR data revealed sharp doublet peaks for Hα and Hβ at 7.71–7.69 and 7.77–7.74, and 7.69–7.66 and 7.82–7.79, respectively. The *trans* conformation of the chalcones' double bond was indicated by a significant coupling constant of 15 Hz^77^. The α, β-unsaturated ketone system was confirmed by the presence of a sharp deshielded sp^2^ carbonyl carbon at 181.42 δ and 187.11 δ in the ^13^C-NMR of **B10** and **B15**, respectively. The molecular weights of the compounds were revealed by the HRMS analysis (Supplementary Material).

### Inhibition studies of hMAO-A and hMAO-B

All fifteen chalcone derivatives (**B1–B15**) showed more effective inhibitory activity against hMAO-B than hMAO-A. Experimentally, most of the compounds showed residual activity of < 50% for hMAO-B at a concentration of 10 μM (Table 1). Compound **B10** most potently inhibited hMAO-B with an IC_50_ value of 0.067 μM, followed by **B15** (IC_50_ = 0.120 μM). Structurally, the benzylated chalcone in which the **A**-ring was substituted with thiophene showed the highest hMAO-B inhibition. Compared with the ethoxy group (**B15**) at the *para* position of the **A**-ring, hMAO-B inhibition of **B10** was 1.79 times higher than **B15**. This means that thiophene substitution in the **A**-ring increases hMAO-B inhibition. In addition, for all compounds, regardless of the **A**-ring, the benzyloxy group at the *para* position of the **B**-ring showed higher hMAO-B inhibition than the *ortho* position. These results indicated that the benzyloxy group at the *para* position of the **B**-ring enhanced MAO-B inhibition. Selectivity index (SI) values for hMAO-B of **B10** and **B15** were calculated as 504.791 and 287.600, respectively, suggesting that **B10** and **B15** are potential selective hMAO-B inhibitors.

**Table 1:** Inhibitions of hMAO-A and hMAO-B by **B** series.

Compounds	Residual activity at 10 µM (%)		IC_50_ (µM)		SI^a^
	hMAO-A	hMAO-B	hMAO-A	hMAO-B	
**B1**	82.62 ± 5.03	35.53 ± 6.05	26.923 ± 4.160	1.441 ± 0.149	18.677
**B2**	53.15 ± 1.58	23.59 ± 0.96	13.728 ± 1.531	0.626 ± 0.296	21.933
**B3**	58.32 ± 0.84	0	14.267 ± 0.203	0.261 ± 0.016	54.663
**B4**	78.25 ± 4.32	16.24 ± 6.49	29.376 ± 2.708	0.233 ± 0.110	125.915
**B5**	89.21 ± 4.47	0	> 40	0.224 ± 0.082	> 178.651
**B6**	92.00 ± 2.93	41.33 ± 4.90	> 40	6.550 ± 1.344	> 6.107
**B7**	58.43 ± 4.48	0	12.597 ± 0.898	0.149 ± 0.006	84.544
**B8**	92.45 ± 0.49	64.36 ± 1.50	> 40	17.880 ± 0.268	> 2.237
**B9**	69.09 ± 1.54	0	23.966 ± 1.443	0.236 ± 0.017	101.551
**B10**	76.67 ± 3.22	0	33.821 ± 6.888	0.067 ± 0.005	504.791
**B11**	81.27 ± 2.92	41.20 ± 0.12	> 40	5.784 ± 2.387	> 6.915
**B12**	86.70 ± 2.68	75.44 ± 3.60	> 40	37.559 ± 1.882	> 1.065
**B13**	99.12 ± 1.24	1.39 ± 3.27	> 40	0.255 ± 0.016	> 156.617
**B14**	91.23 ± 6.20	51.85 ± 1.31	> 40	11.347 ± 0.331	> 3.525
**B15**	75.88 ± 3.10	0	34.512 ± 3.544	0.120 ± 0.010	287.600
Toloxatone			1.080 ± 0.025	–	
Lazabemide			–	0.110 ± 0.016	
Clorgyline			0.007 ± 0.0007	–	
Pargyline			–	0.140 ± 0.0059	

Results are the means ± standard errors of duplicate or triplicate experiments.

^a^Selectivity index (SI) values are expressed for hMAO-B as compared with hMAO-A.

### Kinetic study

**Figure 2:**
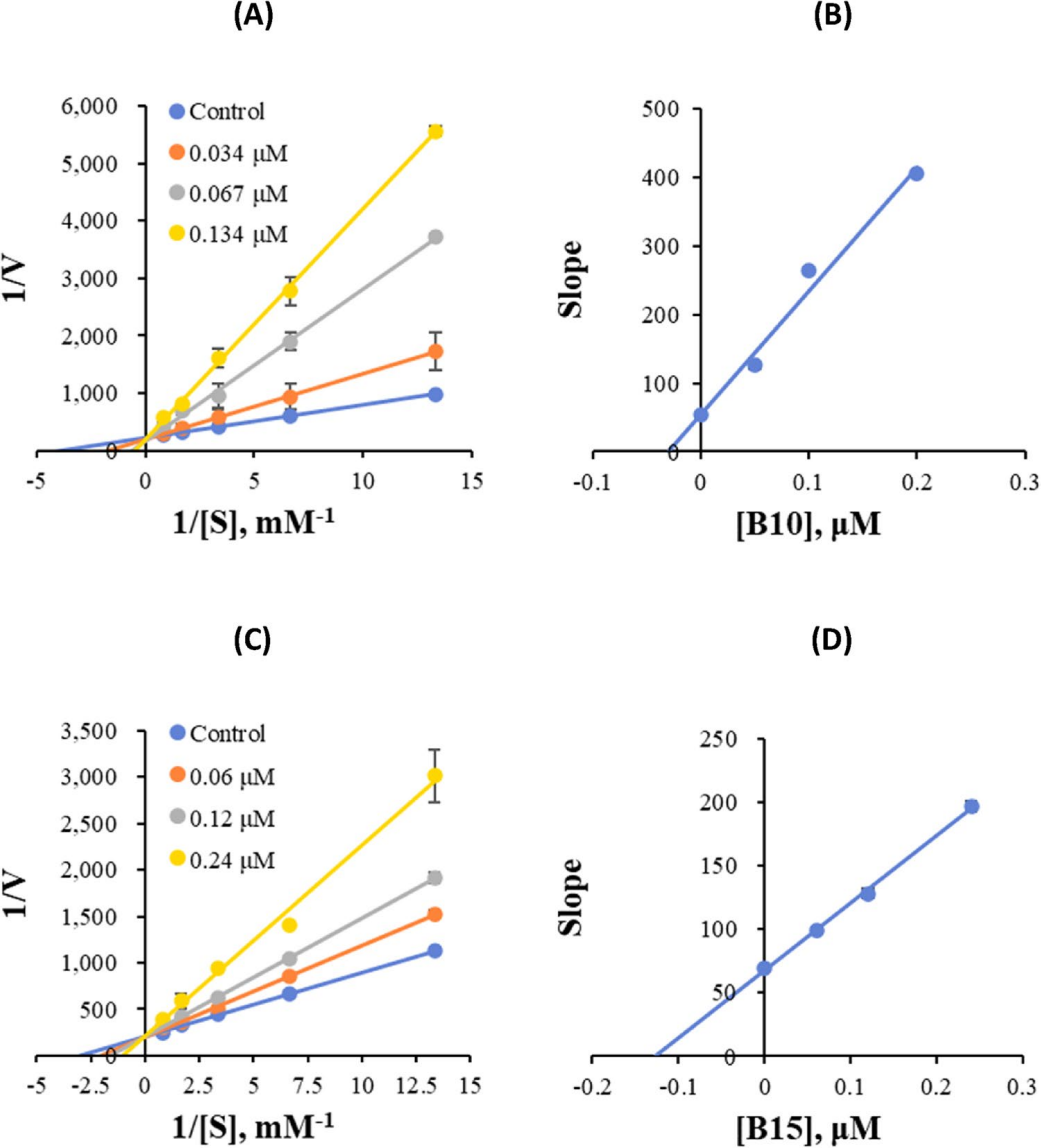
Lineweaver–Burk plots for the inhibition of hMAO-B by **B10** (**A**) and **B15** (**C**), as well as the corresponding secondary plots of the slope vs inhibitor concentration (**B**,**D**, respectively).

Kinetic studies were carried out at five concentrations of the substrates and three inhibitor concentrations. In the kinetic studies of hMAO-B binding by **B10** and **B15**, Lineweaver–Burk plots showed that **B10** and **B15** were competitive inhibitors of hMAO-B (Fig. 2A,C), and their secondary plots showed that their K_i_ values were 0.030 ± 0.001 and 0.033 ± 0.001 μM, respectively (Fig. 2B,D). These results suggested that **B10** and **B15** were competitive with the substrate at the active site of hMAO-B.

### Reversibility studies

**Figure 3:**
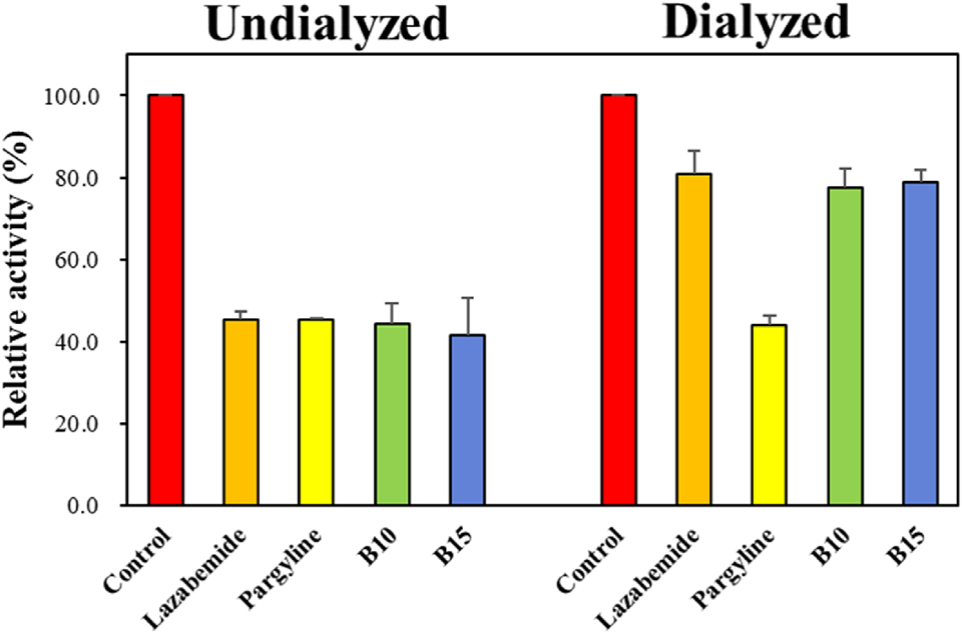
Recoveries of hMAO-B inhibitions by **B10** and **B15** using dialysis experiments.

Upon preincubating hMAO-B with **B10** and **B15** for 30 min, the reversibility of the inhibitors was examined by dialysis. **B10** and **B15** were utilized at concentrations of 0.134 and 0.240 μM in the tests, along with lazabemide (a reference reversible inhibitor) at 0.220 μM and pargyline (a reference irreversible inhibitor) at 0.280 μM. To identify the reversibility patterns, the relative activity of dialyzed (A_D_) was compared to the one of undialyzed (A_U_). The inhibitions of hMAO-B by **B10** and **B15** were recovered from 44.29% (A_U_) to 77.53% (A_D_) and 41.68% (A_U_) to 78.78% (A_D_), respectively, in reversibility tests (Fig. 3). The recovery values of the samples were comparable to lazabemide, a reference inhibitor for hMAO-B that is reversible (from 45.13% to 80.78%), and distinct from pargyline, a reference inhibitor for hMAO-B that is irreversible (from 45.26% to 43.96%). These findings suggested that **B10** and **B15** were hMAO-B reversible inhibitors.

### Structure–activity relationships (SAR)

In the SAR investigation, variously substituted chalcones with phenyl and heterocyclic systems were employed. The study focused primarily on the effects of placing the benzyloxy and different electron-donating and withdrawing groups. When chalcones with a heterocyclic system (**B8–B11**) were compared to those with a phenyl system in terms of their inhibitory profile, the heterocyclic chalcones exhibited relatively higher hMAO-B inhibition. Contrasting the inhibitory values of methylenedioxy (**B1** and **B2**) and benzodioxane (**B3**) chalcones, the latter exhibited greater hMAO-B inhibition than the former, suggesting that increasing the number of alkyl groups between the two oxygen atoms enhanced hMAO-B inhibition as well as selectivity. Furthermore, the hMAO-B inhibitory ranges of the methylenedioxy chalcones (**B1** and **B2**) displayed that compounds with identical attachments appeared to have vastly differing hMAO-B inhibitory potentials, depending on the position of the benzyloxy group, with the relocation of the benzyloxy group from the *ortho* (**B1**) to *para* position (**B2**), reducing the hMAO-B inhibitory value to half and intensifying the SI 1.17 times. The *para* placement of the benzyloxy group in benzodioxane chalcone (**B3**) raised the hMAO-B inhibitory range thrice higher than that of the methylenedioxy chalcone (**B2**) with 2.49 times greater selectivity in conjunction to the increased number of alkyl groups.

Analogues with bromothiophene ring presented the strong hMAO-B inhibiting heterocyclic chalcone, when the benzyloxy group tethered to the *para* position (**B8**). Shifting the benzyloxy group to the *ortho* position (**B9**) exhibited the lowest inhibiting chalcone of the heterocyclic series with an SI difference of 45.39-fold. The greatest hMAO-B inhibitory chalcone (**B10**) of the entire series, with an SI value of 504.791 on anchoring the benzyloxy on *para* position, was shown by analogues with thiophene rings in comparison. In contrast, its *ortho* derivative (**B11**) showed a poor inhibition of hMAO-B and its SI value was 72.99 times lower than that of the *para* derivative. The inhibitory profiles of both bromothiophene (**B8** and **B9**) and thiophene (**B10** and **B11**) analogues showed how the bromine molecule affects the inhibition value of both *para* and *ortho* benzyloxy tethered chalcones, with the *para* analogues (**B8** and **B10**), showing a variation of 4.97-fold and the *ortho* analogues (**B9** and **B11**), expressing a difference of 3.09-fold in SI. Contrary to the benchmarks clorgyline (IC_50_ = 0.110 µM) and pargyline (IC_50_ = 0.140 µM), the highest activity compound (**B10**) had a low IC_50_ value of 0.067 µM.

Similar to this, chalcones with a phenyl system demonstrate a change in IC_50_ value when the position of the benzyloxy group was swapped from *ortho* to *para*. With an SI variation of 1.41-fold, methyl sulfonyl chalcones showed a lower IC_50_ value for *para* benzyloxy linked molecule (**B5**) than *ortho* (**B4**). The same property was also disclosed by thiophenyl chalcones, but with a greater SI variation of 13.84 times for *para* (**B7**) over *ortho* (**B6**). The folder SI for trifluoromethyl chalcones differed by 147.05 for the *para* (**B13**) vs the *ortho* (**B12**) analogues, with the *para* analogue having a lower IC_50_ value of 0.255 µM. In a similar vein, ethoxy chalcone (**B14** and **B15**) had an IC_50_ value for its *para* counterpart (**B15**) of 0.120 µM, which was 81.58 times more selective than its *ortho* analogue (**B14**).

**Figure 4:**
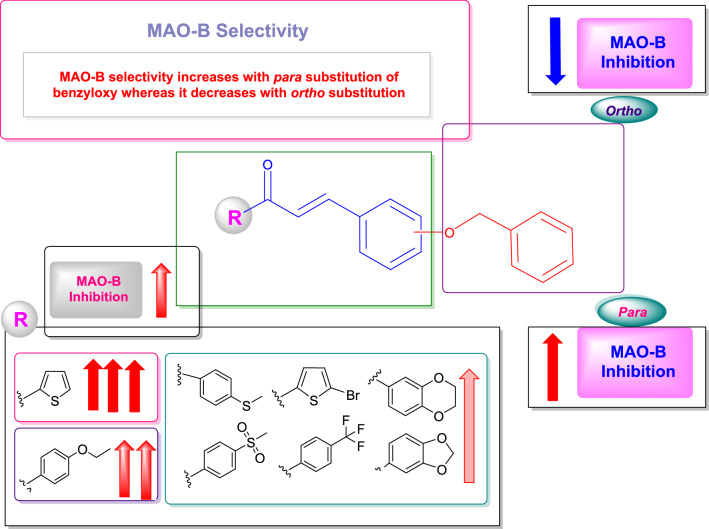
Structure–activity relationships of benzyloxy chalcones.

All these inhibitory profiles of chalcones comprising the phenyl system accentuated that an electron-withdrawing and donating group also influenced the molecules along with the *para* positioning benzyloxy moiety. This highlighted the fact that chalcones with electron-donating groups, such as thiomethyl and ethoxy (**B7**), displayed stronger hMAO-B inhibitory values than chalcones with electron-withdrawing groups, such as methylsulfonyl and trifluoromethyl (**B5** and **B13**). Especially contrasted to the heterocyclic series, ethoxy and thiomethyl chalcones (**B15** and **B7**) exhibited the greatest hMAO-B IC_50_ values. However, the former gave the second-highest IC_50_ value of the whole series (IC_50_ = 0.120 µM), which was greater than that of the reference standard pargyline. According to the SAR, chalcones with *para* versus *ortho* positioned benzyloxy groups, the former had a greater influence. Additionally, the electron-donating group had a more beneficial impact on chalcone activity than the electron-withdrawing group (Fig. 4). The summarized SAR of the present study is depicted in Fig. 4.

### Parallel artificial membrane permeability assay (PAMPA) for blood–brain barrier (BBB) permeation study

PAMPA manifested that the benzyloxy chalcones (**B10** and **B15**) had a significant permeability and CNS bioavailability with *Pe* value higher than 4.0 × 10^–6^ cm/s (Table 2). Brain penetration is acritical need for effective CNS medication delivery. In this study, PAMPA-BBB was used to assess the brain penetration of all derivatives. The rate of permeation was calculated using the equation and the compound's effective permeability (Log *Pe*). Molecules exhibiting a *P*e value of lower than 2.0 × 10^–6^ cm/s were categorized as possibly non-BBB permeable (CNS-), whereas compounds exhibiting a *P*e value of more than 4.0 × 10^–6^ cm/s were designated as potentially permeable (CNS+).

**Table 2:** Blood–Brain Barrier assay of key compounds of benzyloxy chalcones by PAMPA method.

Compounds	Experimental *P*e (× 10^−6^ cm/s)	Prediction
B10	4.93 ± 0.13	CNS +
B15	4.09 ± 0.24	CNS +
Selegiline	5.69 ± 0.04	CNS +

*Pe* (10^−6^ cm/s) > 4.00: CNS + (high permeation); *Pe* (10^−6^ cm/s) < 2.00: CNS − (low permeation ); *Pe* (10^−6^ cm/s) from 2.00 to 4.00: CNS ± (BBB permeation uncertain).

### Absorption, distribution, metabolism, and excretion (ADME) properties

A molecule must exhibit significant biological activity at minimal therapeutic doses, being low in toxic effects, and be effective till the intended result is achieved in order to be considered an efficacious medication. For a superior pharmacokinetic profile, the ADME features of drug prospects are taken into accounts during the process of drug discovery. Using online databases like SwissADME (http://www.swissadme.ch/)^78^ and pkCSM (http://biosig.unimelb.edu.au/pkcsm/), the pharmacokinetic properties were estimated in silico^79^. The benzyloxy chalcones ADME characteristics were listed (Table 3). Gastrointestinal permeability and dissolution measurements were used to assess the absorption of the drug. The solubility of proposed molecules spanned from − 5.90 to − 7.165 in aqueous system and was expressed as the logarithm of molar concentration. The percentage absorption of the compounds was evaluated based on the majority of a drug's absorption by the small intestine when taken orally. Since Caco-2 from sentient colon cancer mimics intestine epithelial cells, it is often possible to anticipate the consumption of oral medicines based on Caco-2 permeability. To achieve superior permeability, the compound must have a *P*_*app*_ value greater than 8 × 10^–6^ cm/s. Oddly, all of the substances had high permeability. Each of the molecules exhibited substantial gastrointestinal absorption, ranging from 90 to 95%. Using a volume of distribution (VDss), fraction unbound, and BBB permeability, the drug's distribution profile was projected. If log VDss > 0.45, it suggests that the drug is distributed more widely in the tissues than in the plasma. In the tissues, each component is dispersed at a moderate to low level. Drug effectiveness measured by fraction bound suggests that it is less bound to blood proteins and is hence free to distribute. Both SwissADME and pkSCM were used to evaluate the BBB permeability. For neurodegenerative therapies, BBB permeability is crucial. The lead chemical **B10** had a log BB value of 0.46, indicating that it may easily pass through the BBB. Molecules with a log BB value of 1 are poorly distributed in the brain. The CNS is thought to be penetrable by compounds with a log PS > − 2, whereas those with a log PS < − 3 are thought to be ineffective. All of the substances tested in this study exhibited CNS penetration, therefore. Every molecule has some sort of interaction with cytochromes, whether it be as an inhibitor or a substrate. The study revealed that all benzyloxy chalcones had a reduced total clearance of − 0.196 to 0.755 logml/min/kg. **B10** and **B15** both exhibited total clearances of − 0.053 and 0.251 logml/min/kg, respectively. All of the compounds had favorable ADME characteristics, rendering them all plausible contender.

**Table 3:** ADME properties of benzyloxy chalcones [**B1–B15**].

Code	Absorption			Distribution				^b^Metabolism	^b^Excretion total clearance (logml/min/kg)
	^a^Log S (log mol/L)	^b^Caco-2 perm. (log P_app_ in 10^−6^ cm/s)	^b^Int. abs. (% Absorbed)	^b^VDss (log L/kg)	^b^Fract. Unb(Fu)	^b^BBB perm. (log BB)	^b^CNS perm. (log PS)		
**B1**	− 6.24	1.083	97.33	− 0.124	0.013	0.106	− 1.272	CYP3A4 substrate CYP1A2, CYP2C19 CYP2C9, CYP3A4 inhibitor	0.123
**B2**	− 5.903	1.104	97.102	− 0.199	0.032	0.158	− 1.239	CYP3A4 substrate CYP1A2 CYP2C19 CYP2C9, CYP3A4 inhibitor	0.061
**B3**	− 5.966	1.104	97.507	− 0.145	0.03	0.168	− 1.226	CYP3A4 substrate CYP2C19 CYP2C9 CYP3A4 inhibitor	0.109
**B4**	− 6.495	1.07	98.279	− 0.391	0.008	− 0.512	− 2.066	CYP3A4 substrate CYP2C19 CYP2C9 CYP3A4 inhibitor	0.755
**B5**	− 6.368	1.087	97.33	− 0.478	0.017	− 0.43	− 2.078	CYP3A4 substrate CYP1A2, CYP2C19 CYP2C9 CYP3A4 inhibitor	0.688
**B6**	− 6.929	1.09	95.925	0.191	0	0.51	− 1.137	CYP3A4 substrate CYP1A2, CYP2C19 CYP2C9 inhibitor	− 0.12
**B7**	− 6.927	1.108	94.976	0.094	0.004	0.496	− 1.132	CYP3A4 substrate CYP1A2, CYP2C19 CYP2C9 inhibitor	− 0.192
**B8**	− 6.691	1.058	93.556	0.359	0	0.414	− 1.18	CYP3A4 substrate CYP1A2 CYP2C19 CYP2C9 CYP3A4 inhibitor	− 0.129
**B9**	− 6.826	1.079	93.328	0.286	0	0.402	− 1.154	CYP3A4 substrate CYP1A2, CYP2C19 CYP2C9, CYP3A4 inhibitor	− 0.196
**B10**	− 6.313	1.837	94.423	0.189	0	0.46	− 1.151	CYP3A4 substrate CYP1A2 CYP2C19 CYP2C9 inhibitor	− 0.053
**B11**	− 6.182	2.083	94.651	0.264	0	0.471	− 1.177	CYP3A4 substrate CYP1A2 CYP2C19 CYP2C9 inhibitor	0.014
**B12**	− 7.165	1.106	94.283	0.123	0	0.494	− 1.054	CYP3A4 substrate CYP3A4 CYP1A2 CYP2C19 inhibitor	0.057
**B13**	− 7.137	1.124	93.334	0.028	0	0.479	− 1.048	CYP3A4 substrate CYP1A2, CYP2C19 CYP2C9 inhibitor	− 0.019
**B14**	− 6.71	1.102	97.133	− 0.079	0.017	− 0.192	− 1.247	CYP3A4 substrate CYP1A2, CYP2C19 CYP2C9 inhibitor	0.328
**B15**	− 6.558	1.119	96.184	− 0.0175	0.035	− 0.11	− 1.242	CYP3A4 substrate CYP1A2, CYP2C19 CYP2C9 inhibitor	0.251

The pharmacokinetic properties were calculated in silico using online databases.

^a^SwissADME (http://www.swissadme.ch/).

^b^pkCSM (http://biosig.unimelb.edu.au/pkcsm/). Molecules with Log BB > 0.3 are considered readily to cross the BBB, while molecules with logBB < − 1 are poorly distributed to the brain. Compounds with log PS > − 2 are considered to penetrate the CNS, while those logPS < − 3 are considered unable to penetrate the CNS. perm., permeability.

### Molecular docking

**Figure 5:**
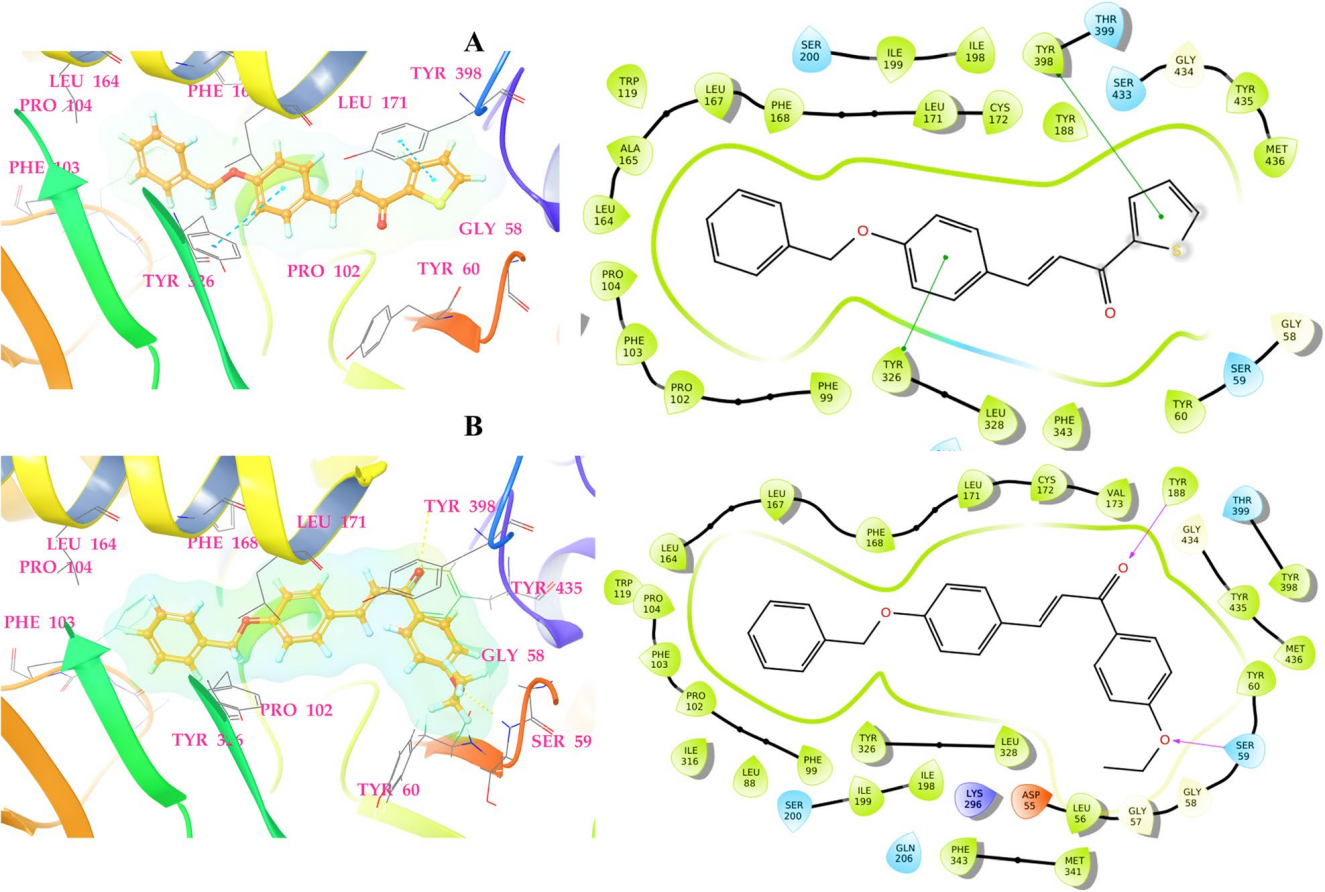
The 2D and 3D binding poses of promising compounds **B10** (**A**) and **B15** (**B**) in the hMAO-B protein binding cavity (PDB ID: 2V5Z).

Compounds **B10** and **B15** were identified to be the most active derivatives in the hMAO-B enzyme inhibition series, as seen in the MAO inhibition assay studies. Accordingly, docking studies were conducted to assess the molecular interactions between the compounds and MAO-B as well as their potential to inhibit the enzyme in silico. The interactions between the compounds and hMAO-B binding pocket are shown in Fig. 5. The SP docking scores for compounds **B10** and **B15** were − 9.954 and − 10.852 kcal/mol, respectively, which were comparable to the cognate ligand (− 10.167 kcal/mol) present in the crystal structure. A detailed analysis of the docking poses of compounds **B10** and **B15** at the active site of hMAO-B revealed that they were located in the 'aromatic enclosure' delineated by Leu64, Leu171, Gly434, Tyr60, Tyr326, Leu328, Pro104, Phe103, Pro102, and Phe99, as illustrated in Fig. 5. The π-π stacking interaction via the phenyl rings of Tyr326 (3.75 Å) and Tyr398 (3.69 Å) with the thiophene and chalcone aromatic rings of **B10,** which is the most important interaction in the putative orientation of the hMAO-B inhibitor. In the case of **B15**, two hydrogen bond interactions with Tyr188 and Ser59 were visible at 3.65 Å and 3.62 Å. The **B15** chalcone aromatic ring unit was buried in a large hydrophobic pocket surrounded by Val173, Cys172, Leu171, Phe168, Leu167, Leu164, Pro104, Phe103, Pro102, Phe99, Tyr326, and Leu328. Through π-π stacking and van der Waals interaction, the benzene ring of both promising compounds interacts with the hydrophobic residue Tyr398 phenyl ring. Interaction with the Tyr398 was required for catalytic activity, and binding of inhibitor candidates in the hMAO-B enzyme's substrate cavity facilitates hMAO-B enzyme inhibition^20^. These data suggest that compounds **B10** and **B15** bind extremely and efficiently to the active site of hMAO-B enzyme.

### MD simulation

The MD simulation is a prominent and popularly used method implemented in recent days in drug development research for enabling the comprehension of energetic details about protein and ligand interactions in a time-affordable fashion. It does this by reproducing the nearly precise or realistic dynamic behavior of a protein–ligand complex. Here, all-atoms classical MD simulations were run for 100 ns on each complex to examine the binding stability at the atomic level and clarify the dynamic properties of the promising hit inhibitors inside the hMAO-B binding cavity. A variety of characteristics from the MD simulation trajectories, including protein backbone RMSD, RMSF, radius of gyration (RGyr), PCA analysis, and binding free energy, were examined in order to assess the stability and flexibility of each protein–ligand complex.

#### Root-mean-square deviation (RMSD)

**Figure 6:**
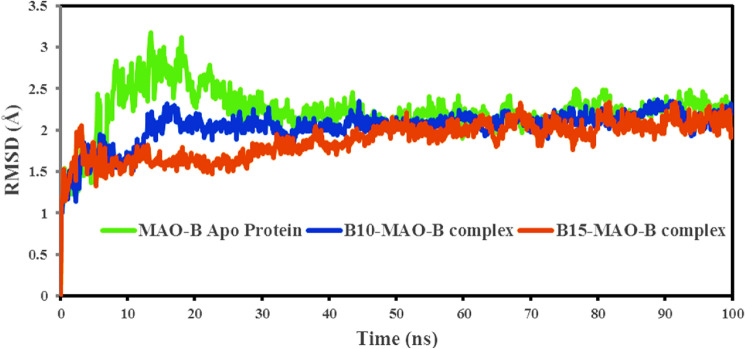
RMSD of the time-dependent hMAO-B protein backbone in complex with compound **B10** (blue) and **B15** (red) during 100 ns simulations.

One of the essential metrics that describes fluctuations in structural conformation of the protein backbone over time during system equilibration is the RMSD value acquired from the MD simulation trajectory, and low and consistent RMSD values show the protein structure's stability. To analyze the ligand–protein interaction, the protein should approach equilibrium, that is, the RMSD value should remain steady^80–82^. Figure 6 shows the RMSD computed for the two complexes based on the protein backbone using the Simulation interaction diagram tool. The average RMSD values were: (a) hMAO-B Apo protein = 2.232 ± 0.29 Å, (b) **B10**-hMAO-B complex = 2.038 ± 0.21 Å and (c) **B15**-MAO-B complex = 2.999 ± 0.22 Å. The RMSD graph for the **B10**-MAO-B complex demonstrated that the initial RMSD increased up to 2.27 Å at 17.5 ns, then stabilized throughout the simulation duration. Due to equilibration, the initial RMSD rose at 3 ns, after which the RMSD ranged from 1.36 to 2.30 Å in the **B15**-hMAO-B complex. The minimal RMSD values clearly indicated that the hMAO-B protein was more conformationally stable when bound with potential inhibitors. There was no significant alteration in hMAO-B backbone RMSD when associated with compounds **B10** and **B15**. By measuring the extent of deviation and comparing with Apo protein, it might be deduced that the hMAO-B protein backbone was bound stably with potential inhibitors.

#### Root-mean-square fluctuation (RMSF)

**Figure 7:**
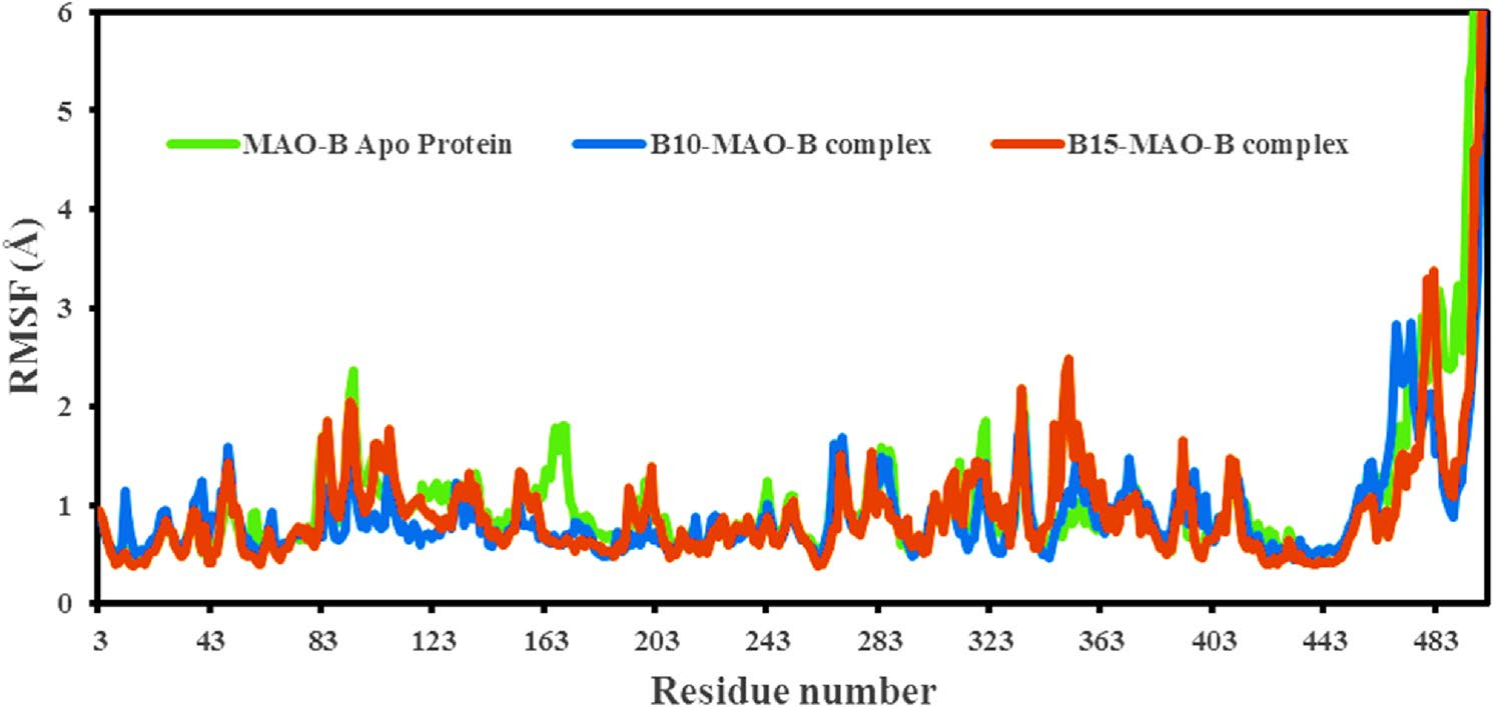
RMSF of the hMAO-B protein backbone in complexes with compound **B10** (blue) and **B15** (red) during 100 ns simulations.

RMSF provides information on the mobility and flexibility of individual amino acids. The lower RMSF suggests less flexibility and mobility of the residue. It could be stated that in ligand–protein interactions, when the RMSF value is low at the active site residues or in residues where the ligand interacts to the protein, the ligand forms a stronger bond with the protein. High RMSF values (peaks) indicated the existence of loops, twists, terminal ends, and loose bonding, i.e., structure flexibility, whereas lower values indicated the presence of secondary structures such as β-sheets and α-helices, i.e., structure stability^83–86^. Compounds **B10** and **B15** complexes' per-residue RMSFs exhibited similar patterns of fluctuating residue involvement, with variations ranging from 0.397 to 2.365 Å; however, compound **B15**'s RMSF values were mildly greater than those of **B10**'s (Fig. 7). During simulation, compound **B10** interacted with 27 amino acids of hMAO-B protein including Thr43 (0.420 Å), Ser59 (0.451 Å), Tyr60 (0.399 Å), Ser59 (0.468 Å), Tyr60 (0.499 Å), Gln65 (0.705 Å), Phe168 (0.587 Å), Leu171 (0.685 Å), Cys172 (0.714 Å), Ile198 (0.602 Å), Ile199 (0.677 Å), Gln206 (0.579 Å), Val294 (0.527 Å), Lys296 (0.464 Å), Ile316 (0.596 Å ), Tyr326 (0.50 Å), Leu328 (0.678 Å), Met341 (0.56 Å), Phe343 (0.481 Å), Leu345 (0.587 Å), Trp388 (0.674 Å), Cys397 (1.101 Å), Tyr398 (1.074 Å), Gly434 (0.617 Å), Tyr435 (0.539 Å), Met436 (0.541 Å), and Ala439 (0.492 Å). All interacting residues in the **B10**-hMAO-B complex had RMSF values of less than 1.10 Å. However, larger fluctuations were identified in the **B10**-hMAO-B complex at residues 469–478 and 496–497, which were near the protein's C-terminus. The **B15** RMSF graph revealed a 4.5 Å RMSF C-terminal area between 480 and 496–497 residual index, indicating a significant structural change in the protein–ligand complex (Fig. 7). **B15** interacted with 23 amino acid residues of hMAO-B, i.e., Thr43 (0.422 Å), Ser59 (0.453 Å), Tyr60 (0.398 Å), Trp119 (0.986 Å), Leu164 (0.65 Å), Leu167 (0.619 Å), Phe168 (0.593 Å), Leu171 (0.667 Å), Cys172 (0.619 Å), Tyr188 (0.491 Å), Ile198 (0.799 Å), Ile199 (0.948 Å), Ser200 (1.116 Å), Gln206 (0.587 Å), Lys296 (0.638 Å), Ile316 (1.215 Å), Tyr326 (0.953 Å), Leu328 (0.856 Å), Phe343 (0.779 Å), Tyr398 (0.486 Å), Gly434 (0.499 Å), Tyr435 (0.441 Å), and Met436 (0.415 Å). The above RMSF results clearly showed that the interacting residues of hMAO-B protein with compounds **B10** and **B15** had small fluctuations, indicating complexes were stable.

#### The radius of gyration (RGyr)

**Figure 8:**
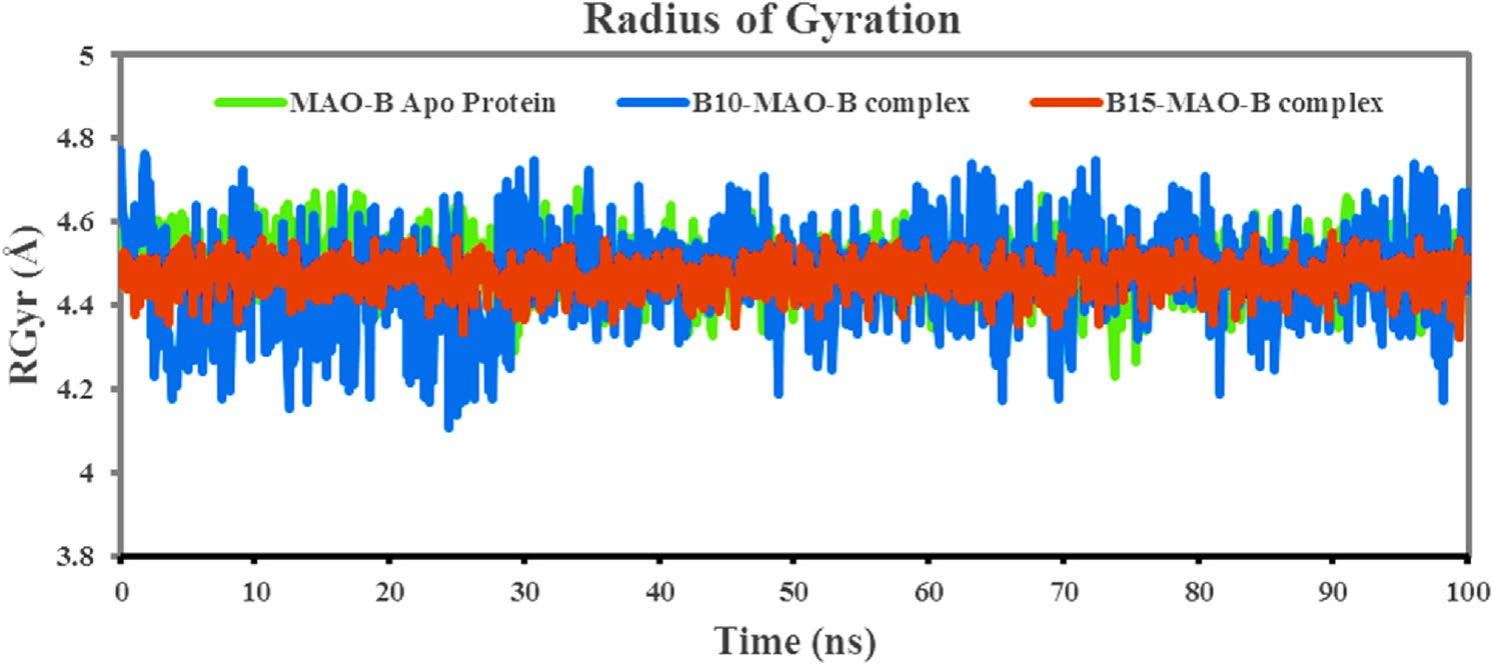
The time-dependent RGyr of **B10**-hMAO-B (blue) and **B15**-hMAO-B (red) complexes during 100 ns simulations.

The radius of gyration (RGyr) determines the compactness or globularity of protein–ligand complexes. An elevated RGyr value generally suggests a more extended or open structural shape of the protein, whereas a lower value indicates a more compact structure^87^. The RGyr values of **B10** and **B15** to hMAO-B were plotted against the time of the simulation (Fig. 8). The Rg vs. time plots for both complexes were quite comparable, and mean values of **B10** (blue) and **B15** (red) with hMAO-B protein were 4.485 and 4.470 Å, respectively, while the value of Apo-hMAO-B protein was 4.493 Å. Compound **B15**'s smaller and more consistent fluctuations than **B10** in Rg value confirmed the prior RMSD finding that the **B15**-hMAO-B complex was stable and compact, resulting in a stronger contact between hMAO-B and ligand. The RGyr values for compound **B10** in the same binding pocket were essentially steady at 4.5 Å from 0 to 28 ns, then ranged between 4.72 and 4.21 Å from 28 to 100 ns. The constant values demonstrated consistent compact behavior.

#### Principle component analysis (PCA)

**Figure 9:**
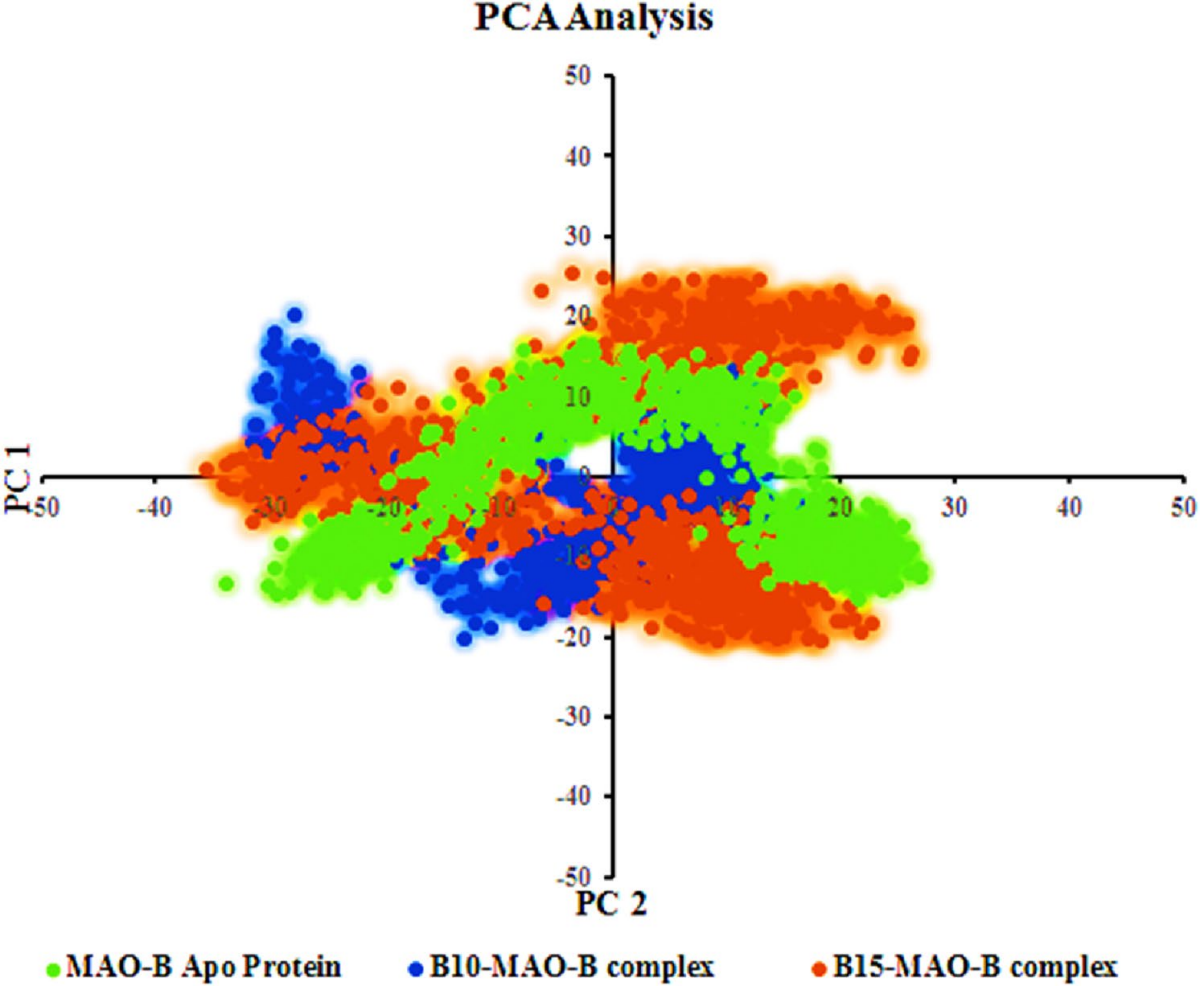
First two eigenvectors describing the protein motion in phase space for **B10**-hMAO-B (blue) and **B15**-hMAO-B (red) complexes.

The PCA method was used to understand conformational distribution during the simulation time and investigate large-scale collective motions of the protein in protein–ligand complexes on the trajectories generated by simulations. The Essential dynamics (ED) analysis script of the Desmond program (*trj_essential_dynamics.py*) was used through the command line for predicting the dynamic behaviors of a protein^88^. This script calculates the principal components of the protein Cα atoms. The complex that occupies less phase space with a stable cluster was assumed to be more stable, whereas the complex that takes up more space with a nonstable cluster was assumed to be less stable^89^. First two principal components (PC1 and PC2) were selected to analyze their projection of trajectories during the simulations of compounds bound to hMAO-B protein in the phase space. The results clearly showed that the drug-protein complexes, **B10**- and **B15**-hMAO-B, occupied smaller regions of phase space (Fig. 9). The trajectories' centering inside a single cluster suggested that MD trajectories moved periodically as a result of steady conformational global motion. The above RMSD, RMSF, RGyr, and PCA parameters derived from the MD simulation trajectories clearly demonstrated that protein–ligand complexes of **B10**- and **B15**-hMAO-B remained stable in dynamic states, and potential hMAO-B inhibitors in the complexes were retained inside the receptor cavity.

#### Binding free energy analysis through MM-GBSA approach

A binding free energy analysis using the MM-GBSA approach was performed on both the protein–ligand complexes to analyze binding affinities of compounds **B10** and **B15** to hMAO-B protein. The MM-GBSA-based binding free energy (∆G_Bind_) computations were performed on the100 ns long MDS trajectories. The binding energies assessed by this method were more efficient and precise, when compared to the binding energies determined in the molecular docking study^90^. The entire trajectories for 100 ns were used for the study, and the average ∆G_Bind_ results are shown in Table 4. The main energy factors used in the calculation of MM-GBSA-based relative binding affinity included the following: lipophilic interaction energy (∆G_Bind_Lipo_), H-bond interaction energy (∆G_Bind_Hbond_), electrostatic solvation free energy (∆G_Bind_Solv_), van der Waals interaction energy (∆G_BindvdW_), covalent interaction energy (∆G_Bind_Cov_), and Coulomb or electrostatic interaction energy (∆G_Bind_Coul_). The Supplementary File (Tables S1 and S2) mentioned the predicted binding energies and contributing factors for the MDS trajectories. It was also revealed that the ∆G_Bind_vdW,_ ∆G_Bind_Lipo_ and the ∆G_Bind_Coul_ energies played a significant role in the ∆G_Bind_ values. Individual investigations indicated that the **B15**-hMAO-B complex had a higher binding energy (∆G_Bind_ = − 87.97 kcal/mol) than the **B10**-hMAO-B complex (∆G_Bind_ = − 75.37 kcal/mol). High non-bonded van der Waals and electrostatic energies could be a component in **B15**'s favorable binding energy. These findings collectively imply that **B10** and **B15** had comparable propensities to stabilize the hMAO-B enzyme.

**Table 4:** The values of RMSD, RMSF, RGyr, and ∆G _Bind_ for **B10**- and **B15**-hMAO-B.

Compounds	RMSD (Å)	RMSF (Å)	RGyr (Å)	Binding energy (kcal/mol)
**hMAO-B Apo protein**				
Minimum	1.065	0.418	4.333	–
Maximum	3.179	9.519	4.658	–
Average	2.232	1.054	4.493	–
**B10-hMAO-B**				
Minimum	1.008	0.451	4.173	− 59.82
Maximum	2.364	7.113	4.740	− 86.59
Average	2.038	0.917	4.485	− 75.37
**B15-hMAO-B**				
Minimum	3.024	0.390	4.323	− 70.82
Maximum	2.937	8.099	4.575	− 101.88
Average	2.999	0.944	4.470	− 87.97

### 2D and 3D QSAR Modeling

#### Estimation of QSARINS-based MLR models

The top-ranked model with the highest statistical significance was further examined for its interpretations, by calculations for internal and external validations. The MLR equation below represents the developed model-9:

pIC_50_ = 4.3121 − 0.1286 * VE3_DzE + 0.0325*TPSA − 1.4362* fr_para_hydroxylation.

*Multivariate models.* **Model 9** (70% training: 30% test set, 3 parametric).

The QSARINS 3 parametric model is currently in development. The descriptor VE3_DzE stands for the logarithmic coefficient sum of the final eigenvector from the Barysz matrix/weighted by Sanderson electronegativities. This description and the activity have negative correlations. The descriptor TPSA stands for the sum of solvent accessible surface areas of atoms having absolute values of partial charges greater than or equal to 0.2. A strong relationship exists between this description and the action. Number of para-hydroxylation sites is represented by the RDKit abbreviation fr_para_hydroxylation. The association between this descriptor and bioactivity is unfavorable.

**Figure 10:**
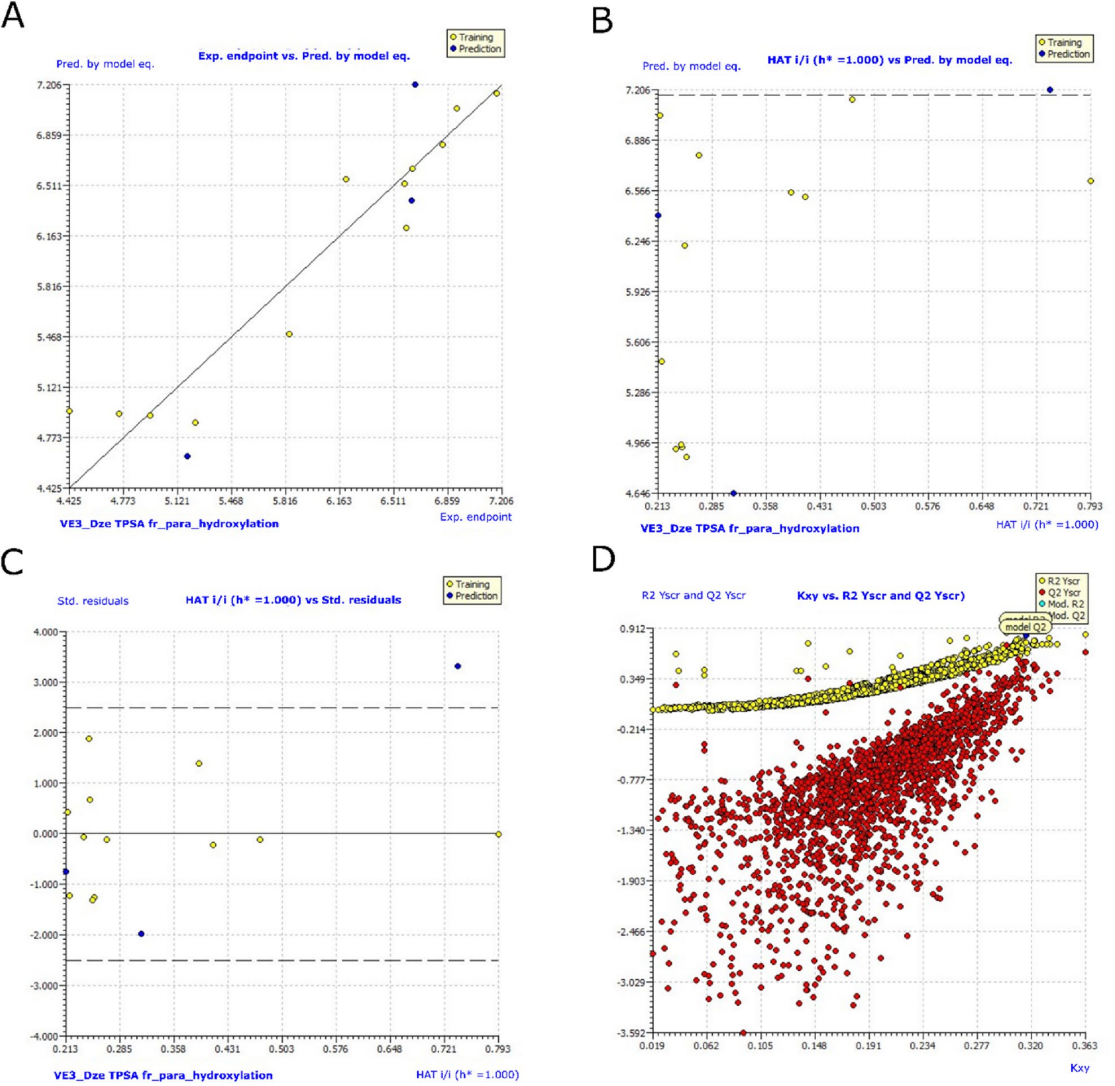
Graphs for model **9**. (**A**) Graph of experimental vs predicted pIC_50_ values; (**B**) Insubria plot; (**C**) William’s plot; (**D**) Y-scrambling plot.

For model **9**, Fig. 10 shows graphs of experimental vs. projected pIC_50_ values, an Insubria plot, a William's plot, a Y-scrambling plot, and an Insubria plot for model **9**. In Table 5, there is additional information on the whole statistical analysis. Additional proof for the GA-MLR QSAR model's statistical robustness was supplied by its various cross-validation qualities (R2 cv, RMSE_cv_, MAE_cv_, CCC_cv_, and Q2 LMO). Greater results for the Tropsha and Golbraikh criterion, Q^2^-F^1^, Q^2^-F^2^, CCC_ex_, and Q^2^-F^3^ demonstrated the external predictive power of the suggested models^91,92^. All models for statics and included descriptors are in Supporting information, Table S3.

**Table 5:** Modelling results for selected QSARINS models with Variable 3 along with their statistical validations.

Statistical parameter	Model-9
**Fitting**	
*R*^*2*^_*tr*_	**0.9125**
*R*^*2*^_*adj*_	0.8797
*R*^*2*^_*tr*_ − *R*^*2*^_*adj*_	0.0328
*LOF*	0.2862
*Kxx*	0.0890
*ΔK*	0.2275
*RMSE*_*tr*_	0.2675
*MAE*_*tr*_	0.2015
*RSS*_*tr*_	0.8586
*CCC*_*tr*_	0.9542
*s*	0.3276
*F*	27.8006
**Internal validation**	
*R*^*2*^_*cv*_ *(Q*^*2*^_*loo*_*)*	**0.8347**
*R*^*2 *^*− R*^*2*^_*cv*_	0.0777
*RMSE*_*cv*_	0.3676
*MAE*_*cv*_	0.2784
*PRESS*_*cv*_	1.6212
*CCC*_*cv*_	0.9144
*Q*^*2*^_*LMO*_	0.7548
*R*^*2*^_*Yscr*_	0.2724
*Q*^*2*^_*Yscr*_	− 0.8777
**External validation**	
*RMSE*_*ex*_	0.4637
*MAE*_*ex*_	0.4365
*PRESS*_*ext*_	0.6450
*R*^*2*^_*ex*_	0.9157
*Q*^*2*^*-F*^*1*^	0.5618
*Q*^*2*^*-F*^*2*^	0.5429
*Q*^*2*^*-F*^*3*^	0.7370
*CCC*_*ex*_	0.8672
*Calc. external data regr. angle from diagonal*	11.1830°
*R*^*2*^*-ExPy (Predictions by LOO)*	0.8372
*R’o*^*2*^	0.8223
*k’*	**0.9984**
*r’*^*2*^_* m*_	0.7352
*R*_*o*_^*2*^	0.8349
*k*	**0.9979**
*r*^*2*^_*m*_	0.7973

*The statistical quality and strength of a GA-MLR based QSAR model was determined on the basis of: (a) internal validation based on leave-one-out (LOO) and leave-many-out (LMO) procedure (i.e. cross-validation (CV)); (b) using external validation; (c) Y-randomization (or Y-scrambling); and (d) fulfilling of respective threshold value for the statistical parameters: ***R***^**2**^_**tr**_** ≥ 0.6, *Q***^**2**^_**loo**_ ≥ **0.5**, *Q*^2^_LMO_ ≥ 0.6, *R*^2^ > *Q*^2^, *R*^2^_ex_ ≥ 0.6, *RMSE*_*tr*_ < *RMSE*_*cv*_, ΔK ≥ 0.05, *CCC* ≥ 0.80, *r*^2^_m_ ≥ 0.6, (1-*r*^2^/*r*_o_^2^) < 0.1, 0.9 ≤ *k* ≤ 1.1 or (1-*r*^2^/*r*’_o_^2^) < 0.1, 0.9 ≤ *k*’ ≤ 1.1,| *r*_o_^2^ − *r*’_o_^2^|< 0.3 with RMSE and MAE close to zero.

Significant values are in bold.

It would be able to identify the reasons for variations in the MAO inhibitory effect of chalcones inhibitors based on benzyloxy pharmacophore by developing QSAR models using a variety of molecular descriptors. Although the current QSAR models have their limitations, more descriptor computation data, accurate modelling, and less statistical artefacts could lead to the development of better models. As a result, each model created here demonstrates the integration of all chosen chemical characteristics and forecasts future pIC_50_ values for the aforementioned analogues.

## Conclusions

Fifteen benzyloxy chalcones (**B1**–**B15**) were synthesized and their effectiveness to inhibit hMAO was evaluated in this study. Notably, contrasted to the reference drugs, the majority of the compounds had a significant selective hMAO-B inhibitory activity. With an IC_50_ value of 0.067 µM, **B10** demonstrated the strongest inhibitory action against hMAO-B, trailed by **B15** (IC_50_ = 0.120 µM). **B10** and **B15** were demonstrated to be competitive and reversible inhibitors of hMAO-B by kinetic and reversibility experiments. In a permeation investigation, **B10** and **B15** exhibited great BBB penetration. Novel insights into the binding modalities of the hMAO-B inhibitor-binding cavity were revealed by MD experiments. In aspects of binding to the hMAO-B enzyme's catalytic domain, both compounds were incredibly potent. Thus, the hMAO-B enzyme was stabilized by **B10**- and **B15**-hMAO-B complexes of higher binding affinities. Additionally, using the descriptors from RDKit and PaDEL, we created a QSAR model. A good balance of external predictive ability was present in the developed QSAR model. The developed model was successful in revealing not only the obvious correlation between structural features but also the hidden correlation between structural features and biological activity. The research also anticipated that introducing halogens to the chalcone framework's benzyloxy pharmacophore could augment MAO-B inhibition. This study, therefore, implies that **B10** and **B15** have therapeutic promise for the treatment of different neurodegenerative illnesses, such as AD and PD. For the lead molecules, in vivo experiments such as hMAO inhibitory activity in cell system including cytotoxicity and neuroprotective activity using OHDA-induced model for PD should be needed in the future study.

## Supplementary Information

Supplementary Information.

## Data Availability

All data generated or analysed during this study are included in this published article and its supplementary information files.
