# Supplementary material for: Introduction of benzyloxy pharmacophore into aryl/heteroaryl chalcone motifs as a new class of monoamine oxidase B inhibitors
Source: Sci Rep. 2022 Dec 27;12:22404. doi: 10.1038/s41598-022-26929-x (PMC9794710; doi:10.1038/s41598-022-26929-x)
Supplement: Supplementary file 1 — Supplementary Information. [file 41598_2022_26929_MOESM1_ESM.docx]

**Supporting Information**

**Introduction of benzyloxy pharmacophore into aryl/heteroaryl chalcone motifs as a new class of monoamine oxidase B inhibitors**

Sachithra Thazhathuveedu Sudevan^a#^, Jong Min Oh^b#^, Mohamed A. Abdelgawad ^c,d^ , Mohammed A.S. Abourehab^e^, T. M. Rangarajan^f^, Sunil Kumar^a^, Iqrar Ahmad^g^, Harun Patel^h^,

Hoon Kim^b*^, Bijo Mathew^a*^

^a^ Department of Pharmaceutical Chemistry, Amrita School of Pharmacy, Amrita Vishwa Vidyapeetham, AIMS Health Sciences Campus, Kochi 682 041, India.

^b^ Department of Pharmacy, and Research Institute of Life Pharmaceutical Sciences, Sunchon National University, Suncheon 57922, Republic of Korea.

^c^ Department of Pharmaceutical Chemistry, College of Pharmacy, Jouf University, Sakaka 72341, Saudi Arabia.

^d^ Pharmaceutical Organic Chemistry Department, Faculty of Pharmacy, Beni-Suef University, Beni-Suef 62514, Egypt.

^e^ Department of Pharmaceutics, College of Pharmacy, Umm Al-Qura University, Makkah 21955, Saudi Arabia.

^f^ Department of Chemistry, Sri Venketeswara College, University of Delhi, New Delhi -110021, India.

^g^ Department of Pharmaceutical Chemistry, Prof. Ravindra Nikam College of Pharmacy, Gondur, Dhule 424002, Maharashtra, India

**^h^** Division of Computer Aided Drug Design, Department of Pharmaceutical Chemistry, R. C. Patel Institute of Pharmaceutical Education and Research, Shirpur 425405, Maharashtra, India

^#^Authors contributed equally.

^*^Corresponding Authors:

Bijo Mathew (B. Mathew) (bijomathew@aims.amrita.edu) (bijovilaventgu@gmail.com)

Hoon Kim (H. Kim) ([hoon@sunchon.ac.kr](mailto:hoon@sunchon.ac.kr))

Table of content

**Figure S1**. ^1^H-NMR spectrum of compound **B1**  3

**Figure S2**. ^13^C-NMR spectrum of compound **B1**  4

**Figure S3**. HRMS of compound **B1**  4

**Figure S4**. ^1^H-NMR spectrum of compound **B2**  5

**Figure S5**. ^13^C-NMR spectrum of compound **B2**  6

**Figure S6**. HRMS of compound **B2**  6

**Figure S7**. ^1^H-NMR spectrum of compound **B3**  7

**Figure S8**. ^13^C-NMR spectrum of compound **B3** 8

**Figure S9**. HRMS of compound **B3** 8

**Figure S10**. ^1^H-NMR spectrum of compound **B4**  9

**Figure S11**. ^13^C-NMR spectrum of compound **B4** 10

**Figure S12**. HRMS of compound **B4** 10

**Figure S13**. ^1^H-NMR spectrum of compound **B6**  11

**Figure S14**. ^13^C-NMR spectrum of compound **B6** 12

**Figure S15**. HRMS of compound **B6** 12

**Figure S16**. ^1^H-NMR spectrum of compound **B7**  13

**Figure S17**. ^13^C-NMR spectrum of compound **B7** 14

**Figure S18**. HRMS of compound **B7** 14

**Figure S19**. ^1^H-NMR spectrum of compound **B8**  15

**Figure S20**. ^13^C-NMR spectrum of compound **B8** 16

**Figure S21**. HRMS of compound **B8** 16

**Figure S22**. ^1^H-NMR spectrum of compound **B10**  17

**Figure S23**. ^13^C-NMR spectrum of compound **B10** 18

**Figure S24**. HRMS of compound **B10** 18

**Figure S25**. ^1^H-NMR spectrum of compound **B11**  19

**Figure S26**. ^13^C-NMR spectrum of compound **B11** 20

**Figure S27**. HRMS of compound **B11** 20

**Figure S28**. ^1^H-NMR spectrum of compound **B14**  21

**Figure S29**. ^13^C-NMR spectrum of compound **B14** 22

**Figure S30**. HRMS of compound **B14** 22

**Figure S31**. ^1^H-NMR spectrum of compound **B15**  23

**Figure S32**. ^13^C-NMR spectrum of compound **B15** 24

**Figure S33**. HRMS of compound **B15** 24

**Table S1.** MM-GBSA based binding free energy for **B10**-MAO-B complex 25

**Table S2.** MM-GBSA based Binding free energy for **B15**-MAO-B complex 28

**Table S3.** Correlation matrix with all QSARINS model 31

**(E)-1-(benzo[d][1,3]dioxol-5-yl)-3-(2-(benzyloxy)phenyl)prop-2-en-1-one [B1] :**^1^H NMR (500 MHz, DMSO) δ: 5.23 (2H, s, -O-CH_2_-C_6_H_5_), 6.16 (2H, s, -O-CH_2_-O-), 7.06-7.01 (2H, Ar-H), 7.24-7.22 (1H, *J* = 10MHz, Ar-H), 7.63-7.40 (8H, Ar-H), 7.88-7.85 (1H, *J* = 15MHz, -CH_α_), 7.95-7.93 (1H, *J* = 10MHz, Ar-H), 8.01-7.98 (1H, *J* = 15MHz, -CH_β_). ^13^C NMR (500 MHz, DMSO) δ: 186.94, 157.39, 151.35, 147.90, 138.33, 136.57, 132.27, 131.89, 129.54, 128.50, 128.03, 127.83, 124.62, 123.14, 121.91, 120.83, 112.92, 107.92, 107.58, 101.94, 69.79. Molecular formula: C_23_H_18_O_4_ (HRMS), Calculated = 358.3866, Observed = 358.3899.


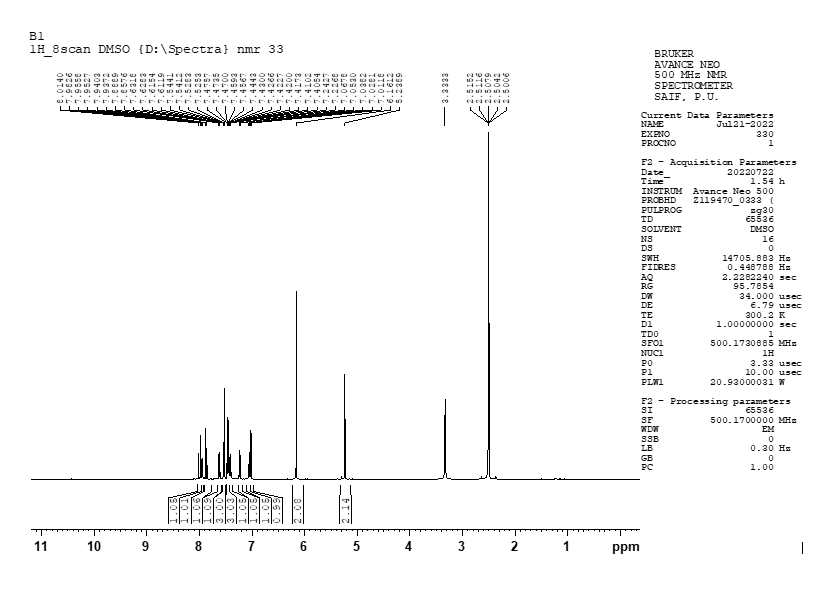
Figure S1 | ^1^H-NMR spectrum of compound **B1.**


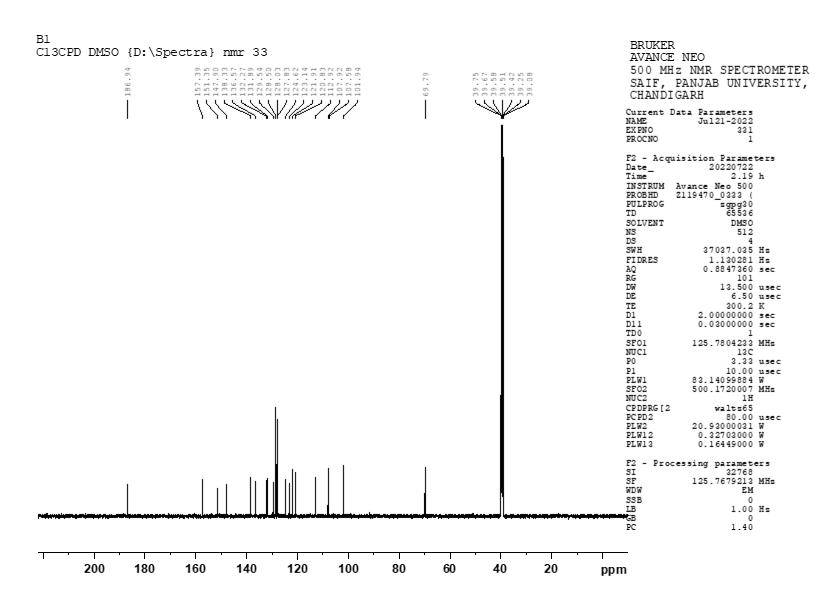
Figure S2 | ^13^C-NMR spectrum of compound **B1.**


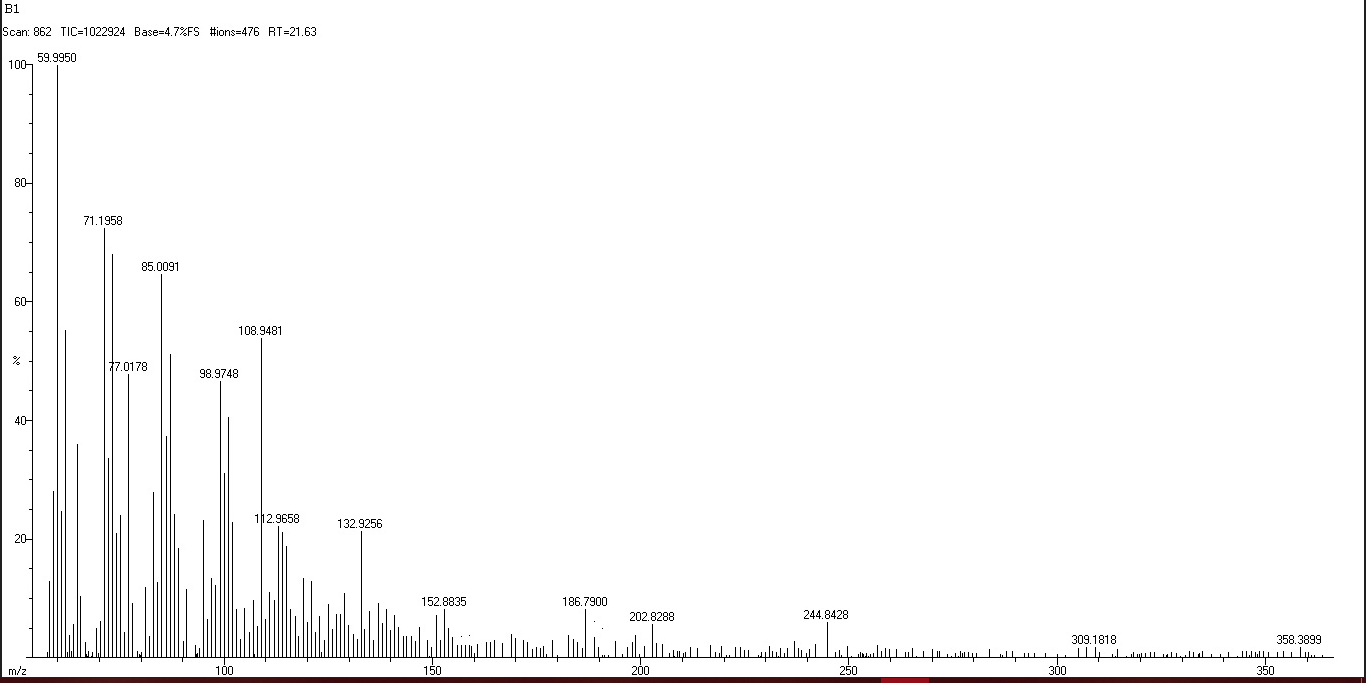


Figure S3 | HRMS of compound **B1.**

**(E)-1-(benzo[d][1,3]dioxol-5-yl)-3-(4-(benzyloxy)phenyl)prop-2-en-1-one [B2] :**^1^H NMR (500 MHz, DMSO) δ: 5.18-5.13 (2H, s, -O-CH_2_-C_6_H_5_), 6.15 (2H, s, -O-CH_2_-O-), 7.06-7.03(1H, *J* = 15MHz, -CH_α_), 7.10-7.08 (2H, *J* = 10MHz, Ar-H), 7.48-7.33 (6H, Ar-H), 7.71-7.64 (2H, Ar-H), 7.80-7.77 (1H, *J* = 15MHz, -CH_β_), 7.85-7.83 (2H, *J* = 10MHz, Ar-H). ^13^C NMR (500 MHz, DMSO) δ: 186.84, 160.28, 151.35, 147.92, 143.31, 136.66, 132.44, 130.67, 128.43, 127.91, 127.72, 127.61, 127.57, 124.82, 119.47, 115.17, 114.84, 108.07, 107.83, 101.97, 72.43, 69.35, 63.03. Molecular formula: C_23_H_18_O_4_ (HRMS), Calculated = 358.3866, Observed = 358.3898.


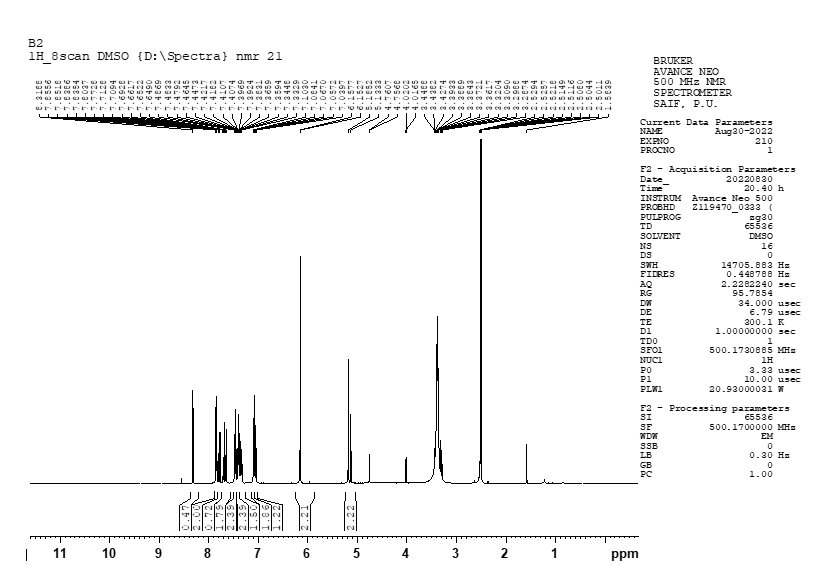


Figure S4 | ^1^H-NMR spectrum of compound **B2.**


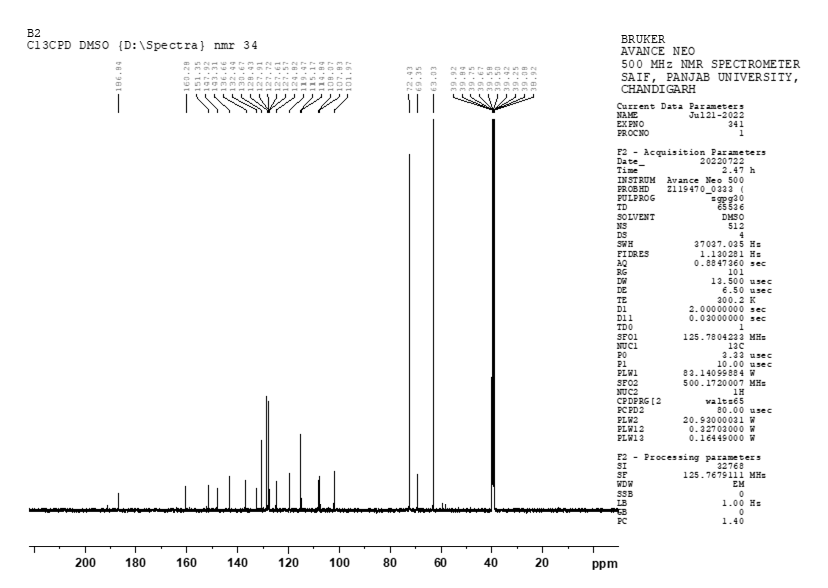


Figure S5 | ^13^C-NMR spectrum of compound **B2.**


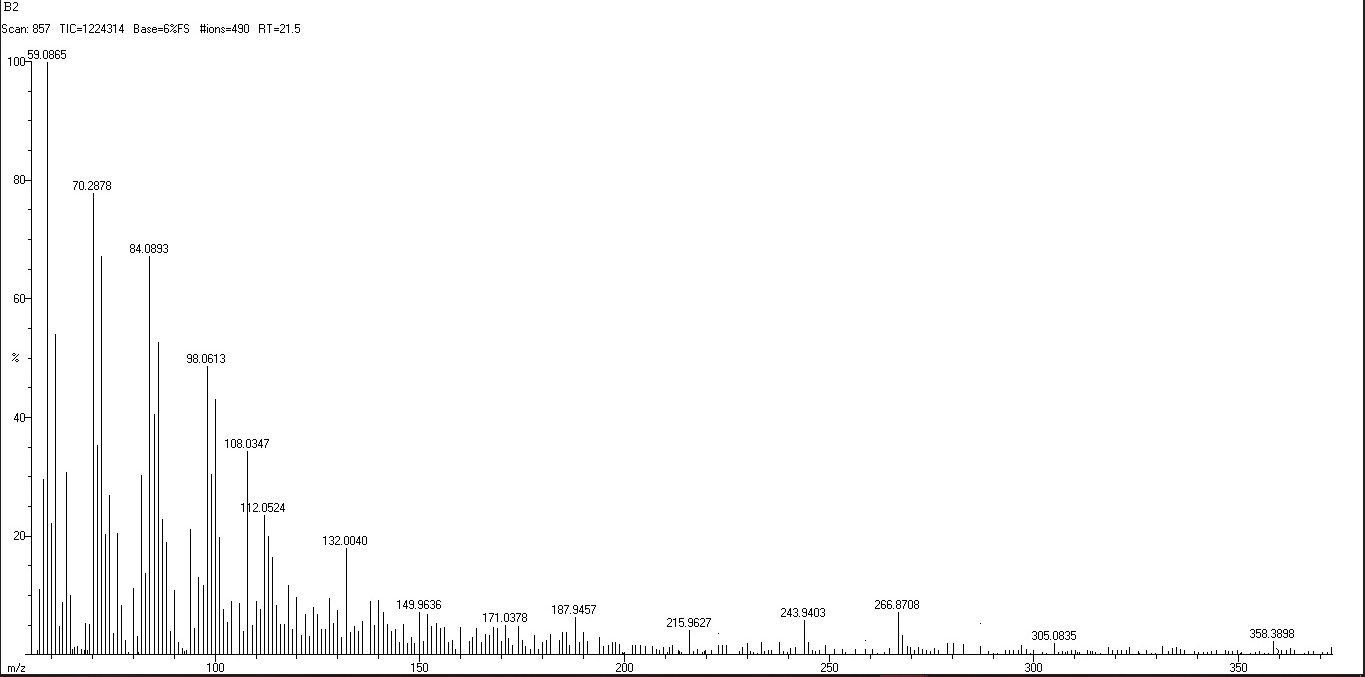


Figure S6 | HRMS of compound **B2.**

**(E)-3-(4-(benzyloxy)phenyl)-1-(2,3-dihydrobenzo[b][1,4]dioxin-6-yl)prop-2-en-1-one [B3] :**^1^H NMR (500 MHz, DMSO) δ: 4.35-4.30 (4H, t, -O-CH_2_-CH_2_-O), 5.18 (2H, s, -O-CH_2_-C_6_H_5_), 7.01-6.99 (1H, Ar-H), 7.09-7.07 (2H, *J* = 10MHz, Ar-H), 7.35-7.34 (1H, Ar-H), 7.40-7.39 (2H, Ar-H), 7.48-7.47 (2H, Ar-H), 7.70-7.68 (2H, *J* = 10 MHz, Ar-H), 7.71-7.68 (1H, *J* = 15MHz, -CH_α_), 7.79-7.76 (1H, *J* = 15MHz, -CH_β_), 7.85-7.83 (2H, *J* = 10MHz, Ar-H). ^13^C NMR (500 MHz, DMSO) δ: 186.94, 160.18, 147.68, 143.21, 143.11, 136.61, 131.29, 130.60, 128.34, 127.81, 127.64, 127.52, 127.37, 119.35, 117.35, 117.00, 115.07, 79.05, 69.27, 64.44, 63.82. Molecular formula: C_25_H_22_O_3_ (HRMS), Calculated = 372.4132, Observed = 370.4498.


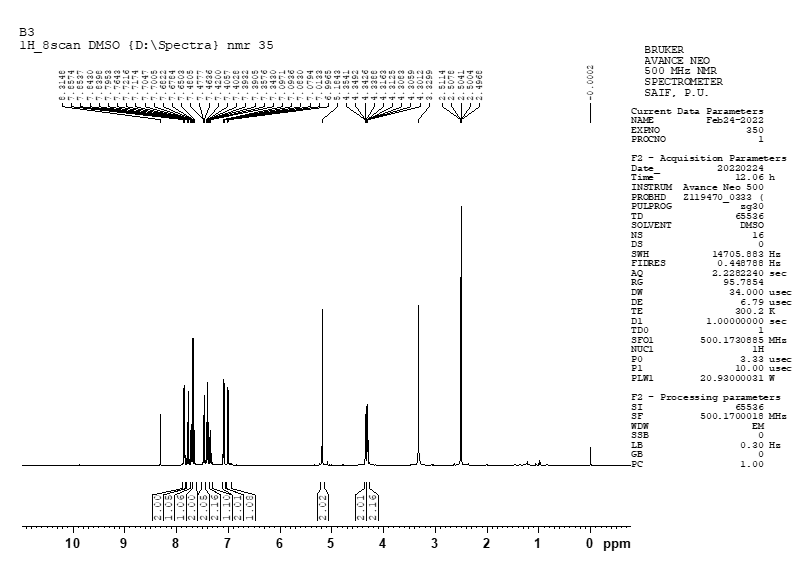
Figure S7 | ^1^H-NMR spectrum of compound **B3.**
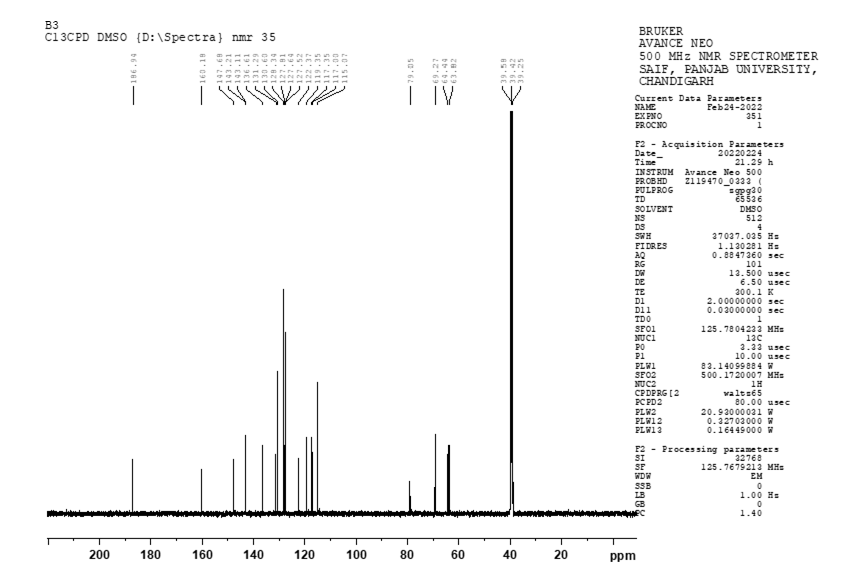
 Figure S8 | ^13^C-NMR spectrum of compound **B3.**


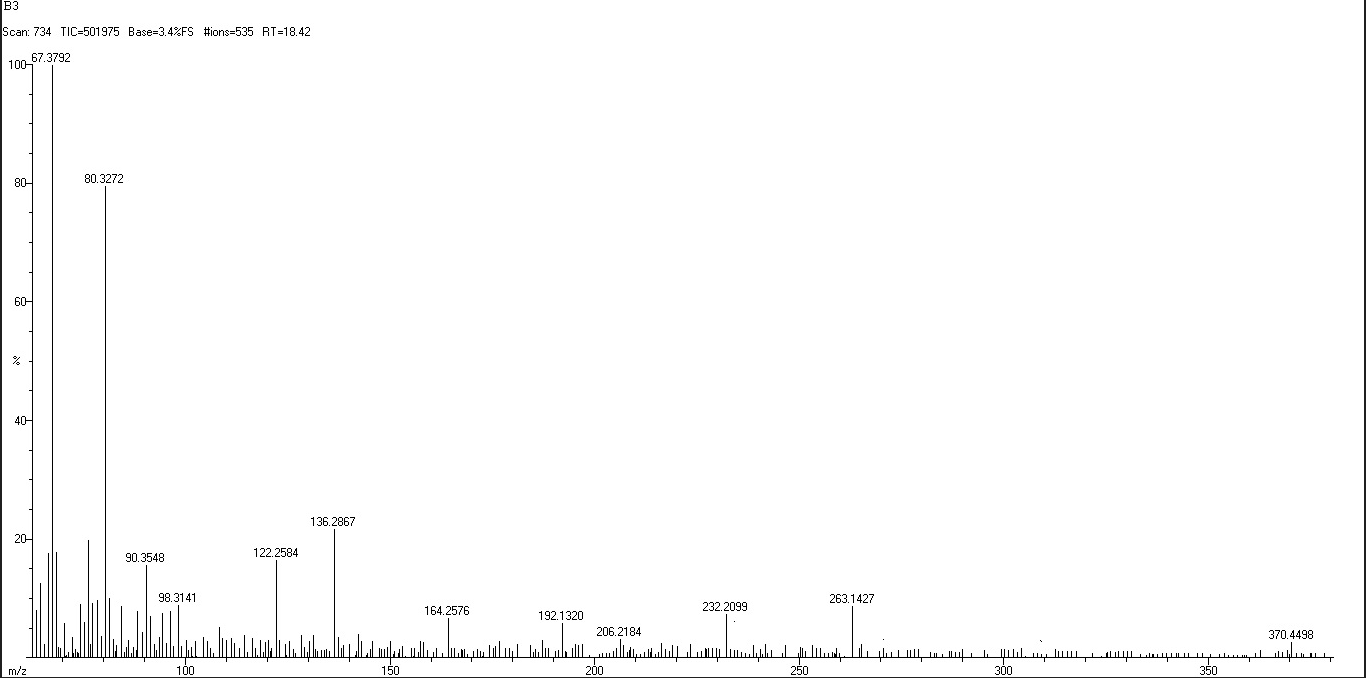


Figure S9 | HRMS of compound **B3.**

**(E)-3-(2-(benzyloxy)phenyl)-1-(4-(methylsulfonyl)phenyl)prop-2-en-1-one [B4] :**^1^H NMR (500 MHz, DMSO) δ: 3.32 (3H,s, -CH_3_-SO_2_-), 5.25 (2H, s, -O-CH_2_-C_6_H_5_), 7.08-7.05 (1H, 15MHz, Ar-H), 7.26-7.24 (1H, *J* = 10MHz, Ar-H), 7.53-7.28 (6H, Ar-H), 7.90-7.87 (1H, *J* = 15MHz, -CH_α_), 7.99-7.96 (1H, *J* = 15MHz, -CH_β_), 8.07-8.05 (3H, *J* = 10MHz, Ar-H), 8.19-8.17 (2H, *J* = 10MHz, Ar-H). ^13^C NMR (500 MHz, DMSO) δ: 188.73, 157.60, 143.92, 141.44, 140.19, 136.53, 132.54, 129.60, 129.01, 128.51, 128.00, 127.81, 127.24, 122.81, 121.91, 120.92, 113.07, 69.84, 43.12, 39.59, 39.42, 39.25. Molecular formula: C_25_H_20_O_4_S (HRMS), Calculated = 392.4675, Observed = 392.4699.


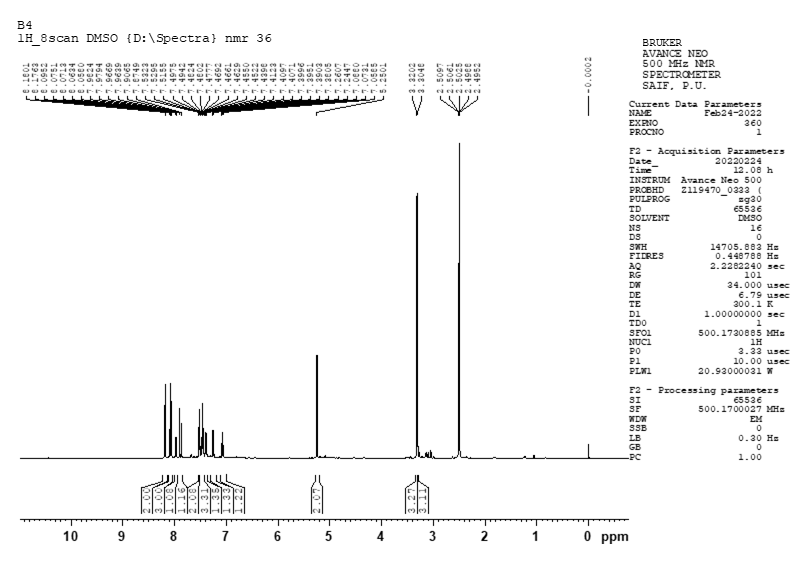
Figure S10 | ^1^H-NMR spectrum of compound **B4.**


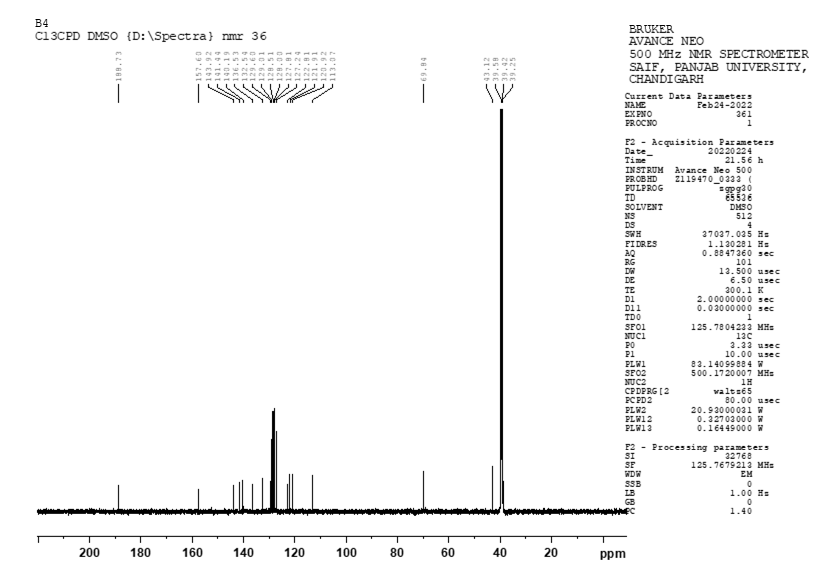
Figure S11| ^13^C-NMR spectrum of compound **B4.**


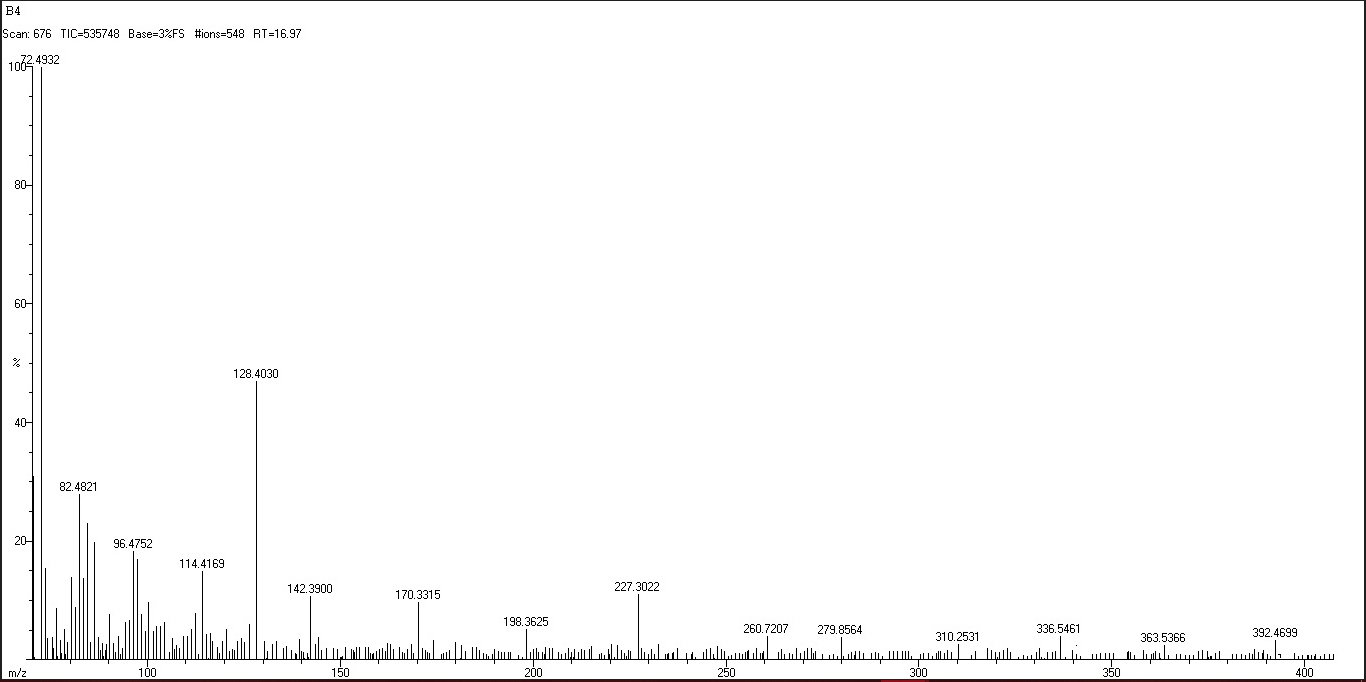


Figure S12 | HRMS of compound **B4.**

**(E)-3-(2-(benzyloxy)phenyl)-1-(4-(methylthio)phenyl)prop-2-en-1-one [B6] :**^1^H NMR (500 MHz, DMSO) δ: 2.56 (3H, s, CH_3_-S-), 5.23 (2H,s,-O-CH_2_-C_6_H_5_-), 7.07-7.04 (1H, *J* = 15MHz, -CH_α_), 7.24-6.23 (1H, Ar-H), 7.35-7.33 (2H, *J* = 10 MHz, Ar-H), 7.47-7.41 (4H, Ar-H), 7.54-7.52 (2H, *J* = 10 MHz, Ar-H), 7.93-7.86 (4H, *J* = 10 MHz, Ar-H), 8.01-7.98 (1H, *J* = 15MHz, -CH_β_). ^13^C NMR (500 MHz, DMSO) δ: 187.93, 157.46, 138.68, 136.56, 133.73, 131.96, 129.70, 128.68, 128.51, 128.01, 127.90, 124.80, 123.09, 122.02, 120.85, 112.92, 69.81, 39.58, 39.42, 39.25, 13.83. Molecular formula: C_23_H_20_O_2_S (HRMS), Calculated = 360.4687, Observed = 360.4698.


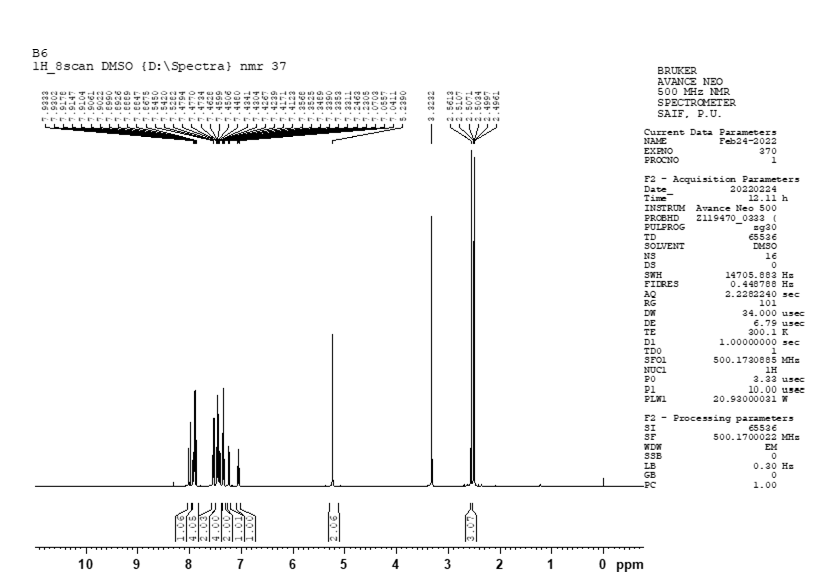
Figure S13 | ^1^H-NMR spectrum of compound **B6**


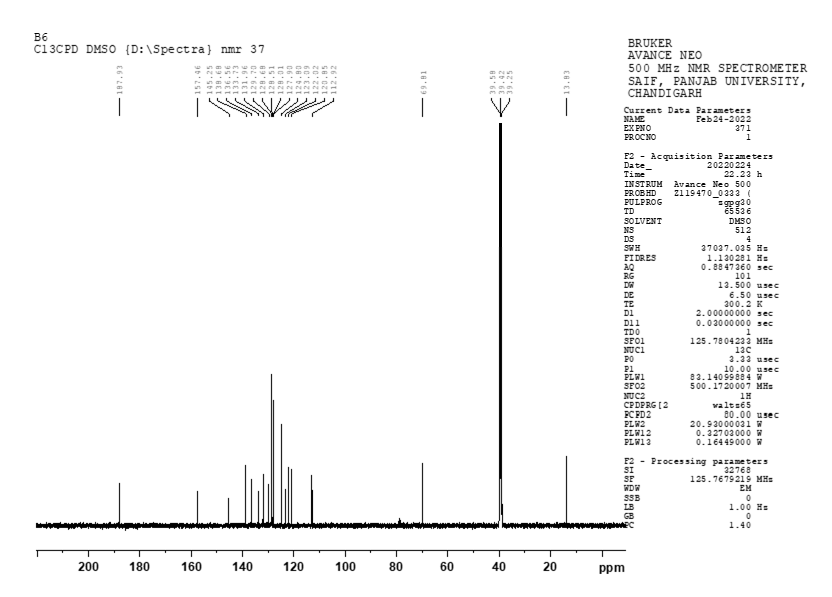
Figure S14 | ^13^C-NMR spectrum of compound **B6.**


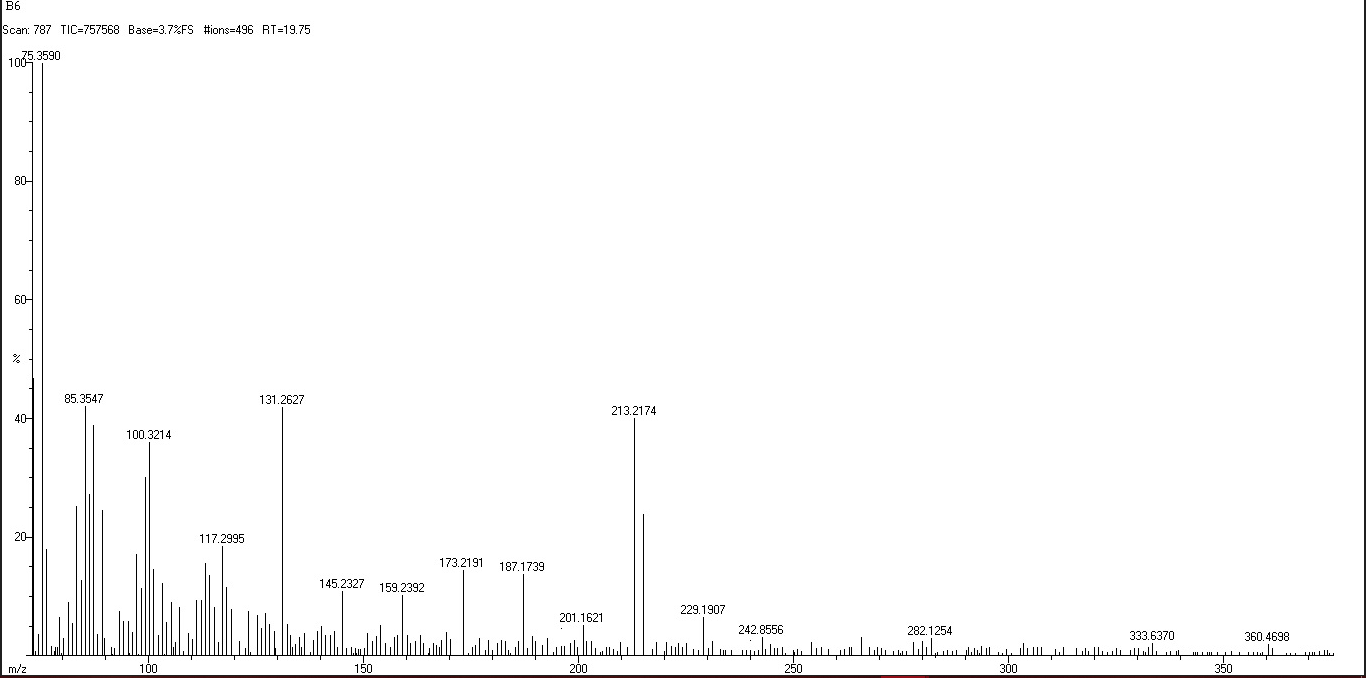


Figure S15 | HRMS of compound **B6.**

**(E)-3-(4-(benzyloxy)phenyl)-1-(4-(methylthio)phenyl)prop-2-en-1-one [B7] :**^1^H NMR (500 MHz, DMSO) δ: 2.56 (3H, s, CH_3_-S-), 5.19 (2H,s,-O-CH_2_-C_6_H_5_-), 7.11-7.09 (2H, *J* = 10 MHz, Ar-H), 7.22-7.20 (1H, *J* = 10 MHz, Ar-H), 7.36-7.34 (1H, *J* = 10 MHz, Ar-H), 7.42-7.39 (4H, *J* = 15 MHz, Ar-H), 7.48-7.46 (2H, *J* = 10 MHz, Ar-H), 7.72-7.69 (1H, *J* = 15MHz, -CH_α_), 7.82-7.79 (1H, *J* = 15MHz, -CH_β_), 7.88-7.84 (2H, Ar-H), 8.10-8.08 (2H, *J* = 10 MHz, Ar-H). ^13^C NMR (500 MHz, DMSO) δ: 187.66, 160.27, 145.12, 143.46, 136.19, 133.88, 131.67, 130.63, 128.84, 128.39, 128.35, 127.95, 127.82, 127.73, 127.73, 127.65, 127.46, 124.82, 115.16, 115.11, 69.54, 13.83. Molecular formula: C_23_H_20_O_2_S (HRMS), Calculated = 360.4687, Observed = 360.4699.


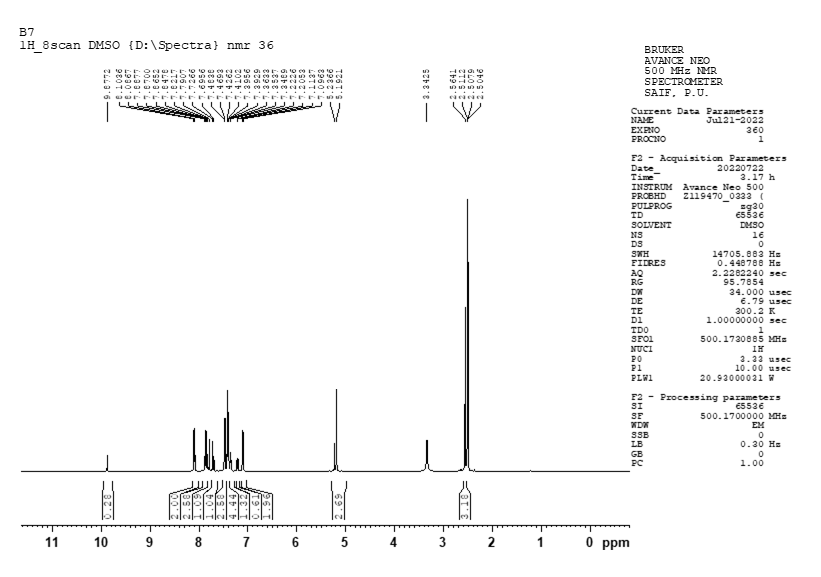
Figure S16 | ^1^H-NMR spectrum of compound **B7.**

**
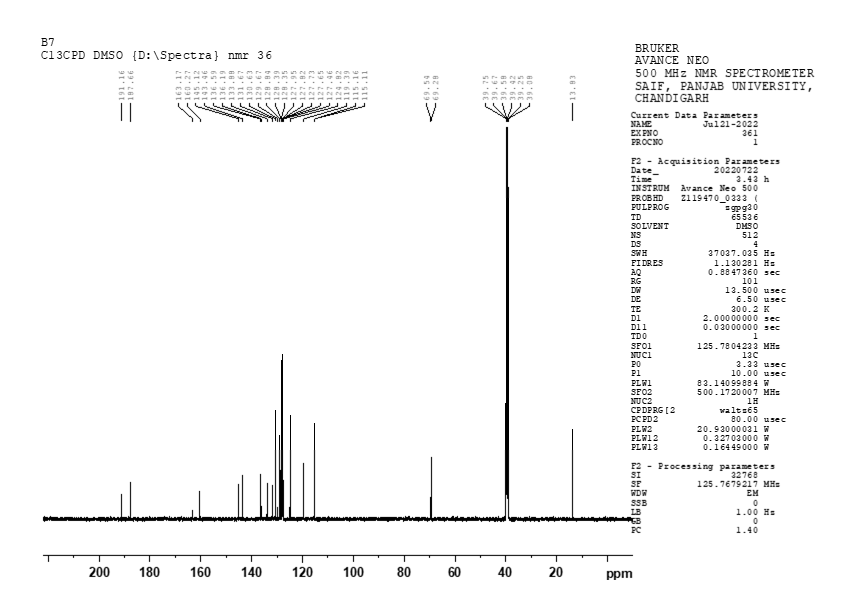
**

Figure S17 | ^13^C-NMR spectrum of compound **B7.**

**
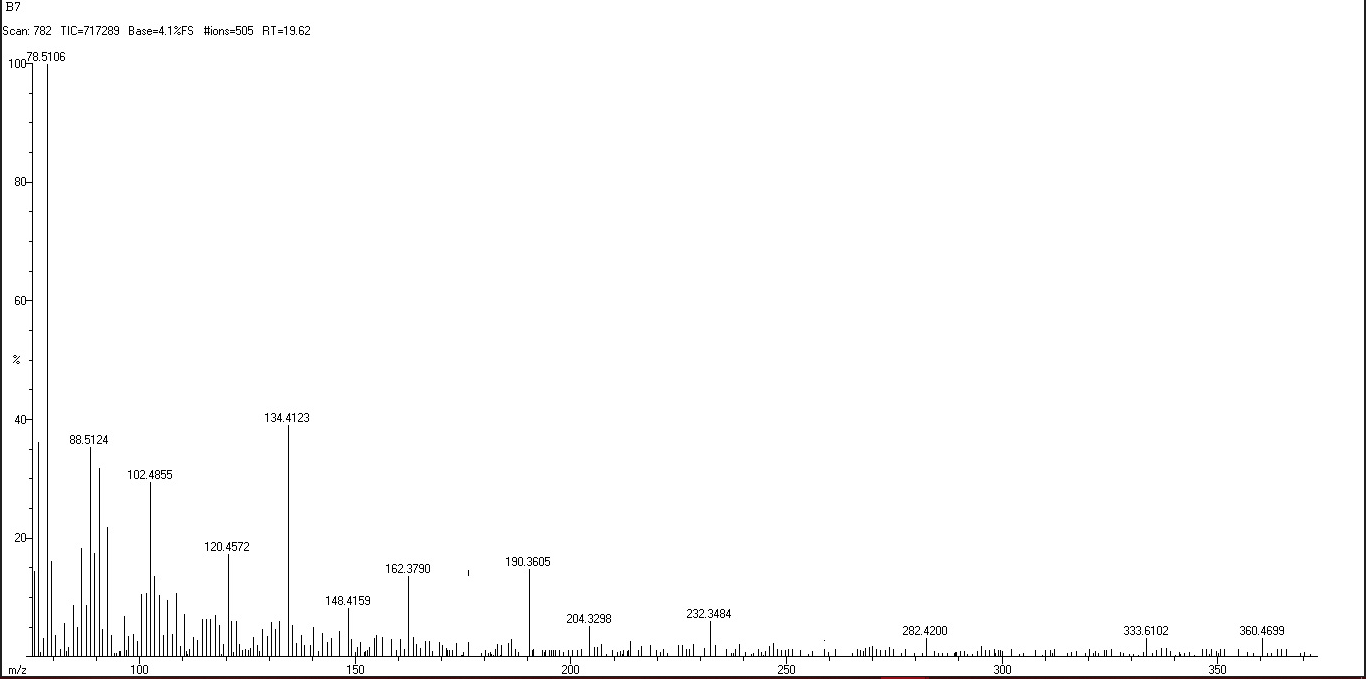
**

Figure S18 | HRMS of compound **B7.**

**(E)-3-(2-(benzyloxy)phenyl)-1-(5-bromothiophen-2-yl)prop-2-en-1-one [B8] :**^1^H NMR (500 MHz, DMSO) δ: 5.24 (2H, s, -O-CH_2_-C_6_H_5_-), 7.07-7.04 (1H, *J* = 15MHz, -CH_α_), 7.24-7.23 (1H, Ar-H), 7.53-7.40 (7H, Ar-H), 7.77 (1H, Ar-H), 7.81-7.80 (1H, Ar-H), 7.95-7.93 (1H, *J* = 10 MHz, Ar-H), 8.02-7.99 (1H, *J* = 15MHz, -CH_β_). ^13^C NMR (500 MHz, DMSO) δ: 180.62, 157.54, 147.11, 138.56, 136.54, 133.55, 132.34, 129.56, 129.00, 128.53, 128.06, 127.89, 122.72, 122.03, 120.92, 120.84, 113.01, 69.81. Molecular formula: C_20_H_15_BrO_2_S (HRMS), Calculated = 399.3009, Observed = 399.2999.


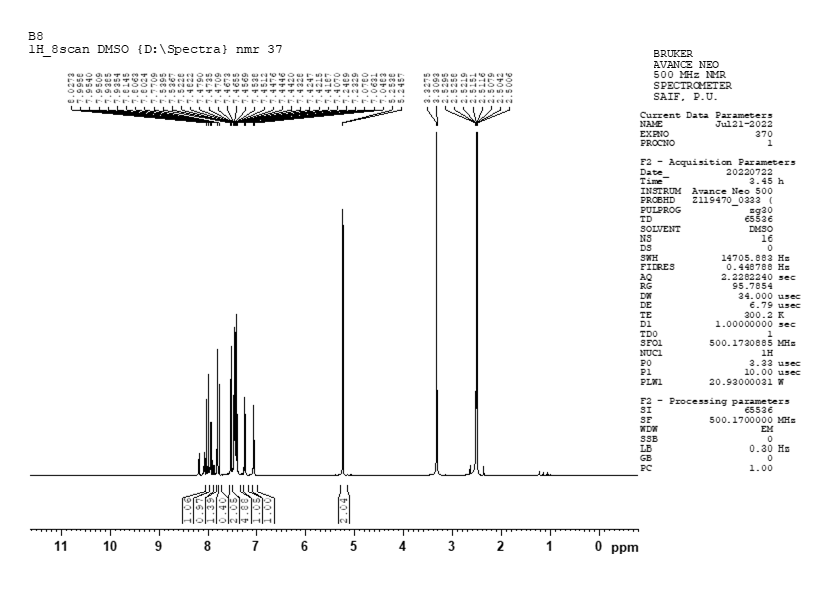
Figure S19 | ^1^H-NMR spectrum of compound **B8.**


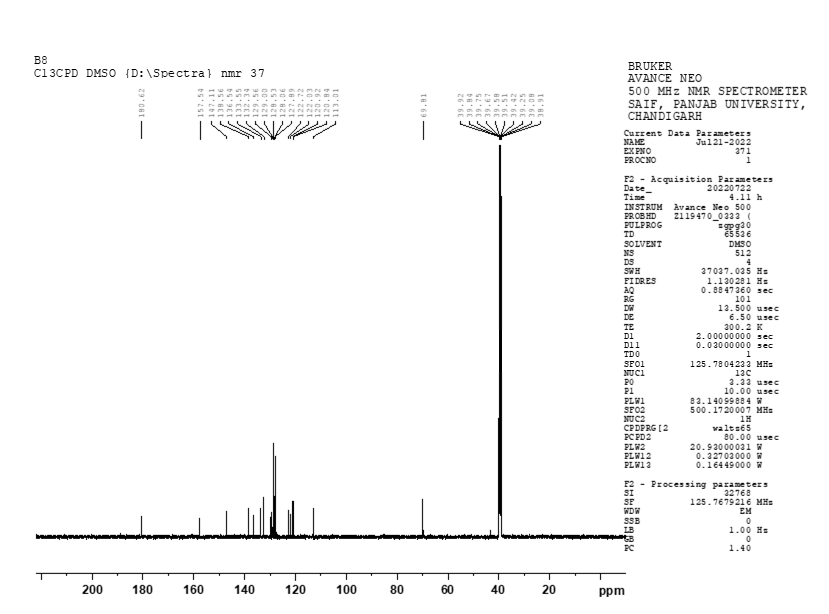


Figure S20 | ^13^C-NMR spectrum of compound **B8.**


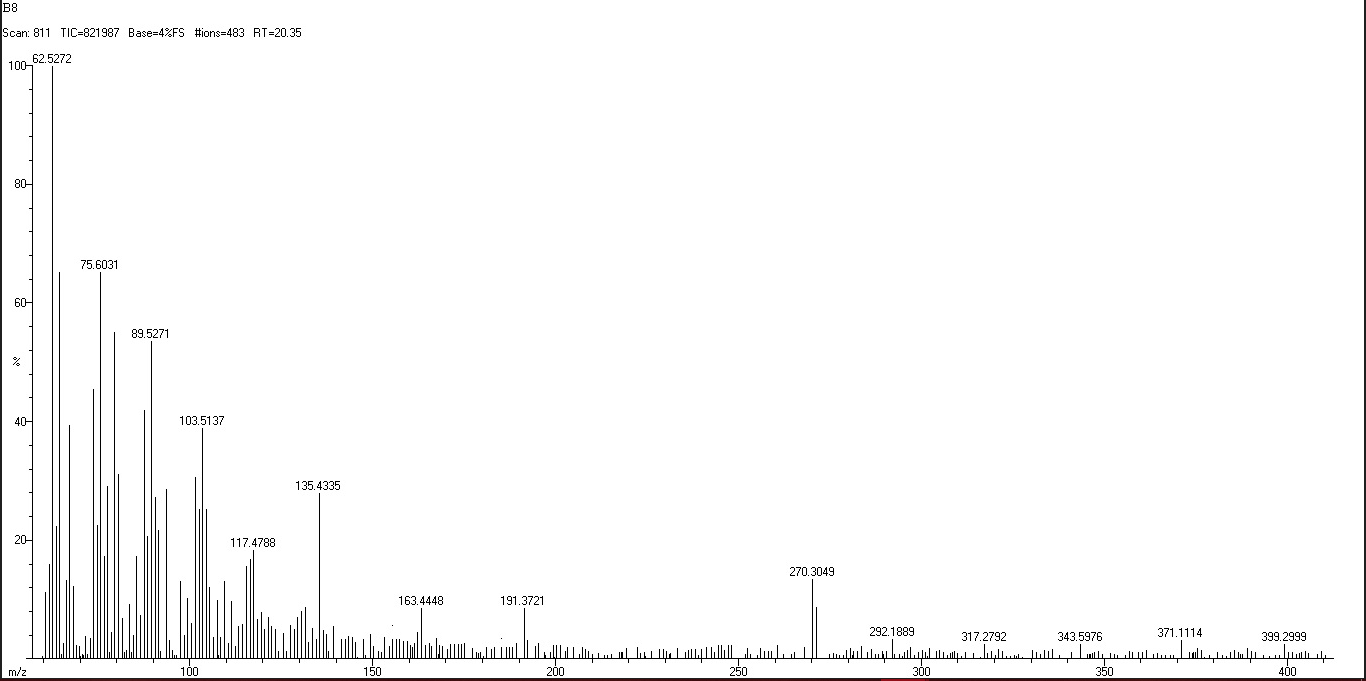


Figure S21 | HRMS of compound **B8.**

**(E)-3-(4-(benzyloxy)phenyl)-1-(thiophen-2-yl)prop-2-en-1-one [B10] :**^1^H NMR (500 MHz, DMSO) δ: 5.19 (2H, s,-O-CH_2_-C_6_H_5_-),7.12-7.10 (2H, *J* = 10 MHz, Ar-H), 7.36-7.30 (2H, Ar-H), 7.48-7.39 (4H, Ar-H), 7.71-7.68 (1H, *J* = 15MHz, -CH_α_), 7.77-7.74 (1H, *J* = 15MHz, -CH_β_), 7.86-7.85 (2H, Ar-H), 8.04-8.03 (1H, Ar-H), 8.30-8.29 (1H, Ar-H). ^13^C NMR (500 MHz, DMSO) δ: 181.42, 160.37, 145.66, 142.92, 136.57, 135.05, 133.13, 130.69, 128.72, 128.35, 127.84, 127.67, 127.23, 119.40, 115.13, 69.30. Molecular formula: C_20_H_16_O_2_S (HRMS), Calculated = 320.4048, Observed =320.4098.


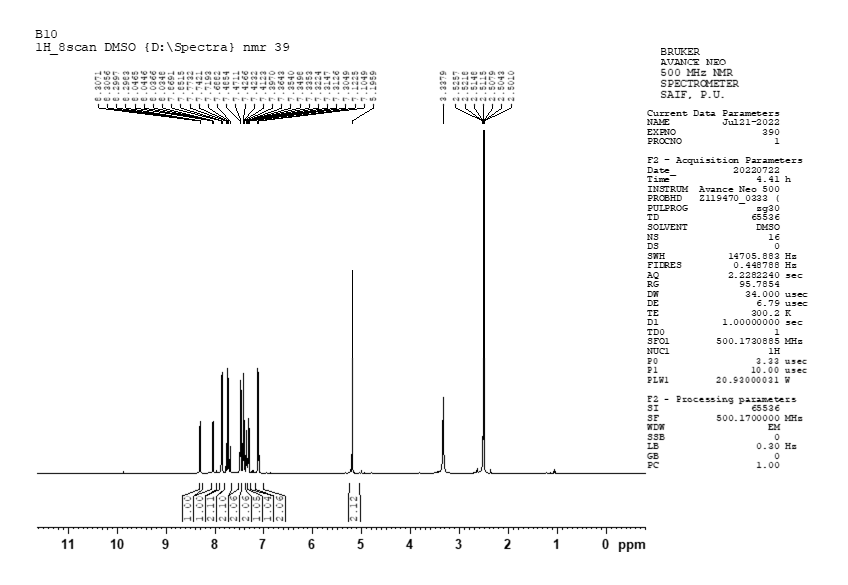
 Figure S22 | ^1^H-NMR spectrum of compound **B10.**


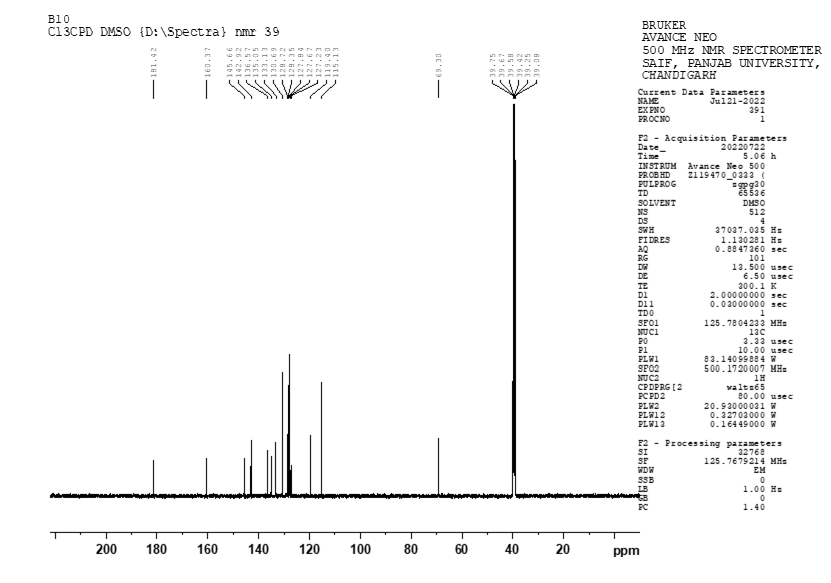


Figure S23 | ^13^C-NMR spectrum of compound **B10.**


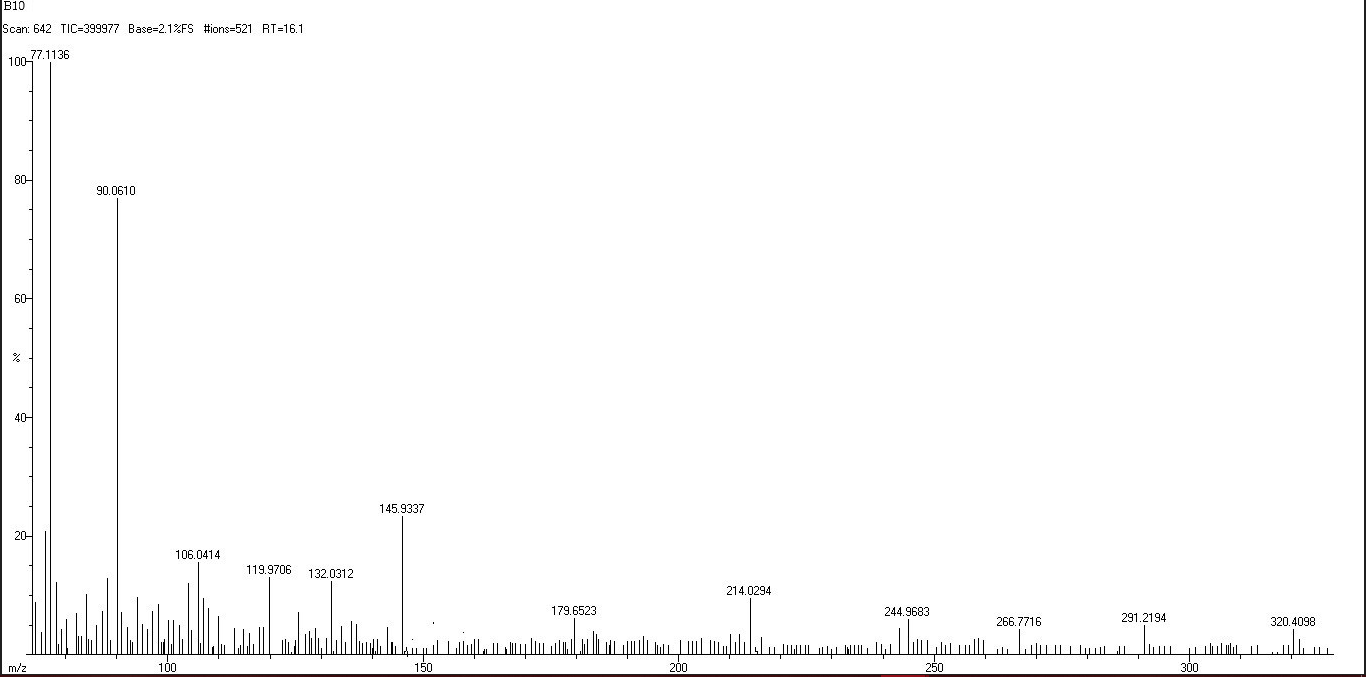


Figure S24 | HRMS of compound **B10.**

**(E)-3-(2-(benzyloxy)phenyl)-1-(thiophen-2-yl)prop-2-en-1-one [B11] :**^1^H NMR (500 MHz, DMSO) δ: 5.24 (2H, s,-O-CH_2_-C_6_H_5_-), 7.07-7.06 (1H, Ar-H), 7.26-7.23 (2H, Ar-H), 7.54-7.40 (6H, Ar-H), 7.85-7.82 (1H, *J* = 15MHz, -CH_α_),7.95-7.92 (2H, *J* = 10 MHz, Ar-H), 7.99 (1H, -CH_β_), 8.03-8.02 (1H, Ar-H). ^13^C NMR (500 MHz, DMSO) δ: 181.60, 157.49, 143.84, 138.02, 136.57, 135.25, 132.2, 129.60, 128.67, 128.52, 128.30, 128.03, 127.89, 127.33, 122.85, 120.85, 112.98, 69.81. Molecular formula: C_20_H_16_O_2_S (HRMS), Calculated = 320.4048, Observed = 320.4098.


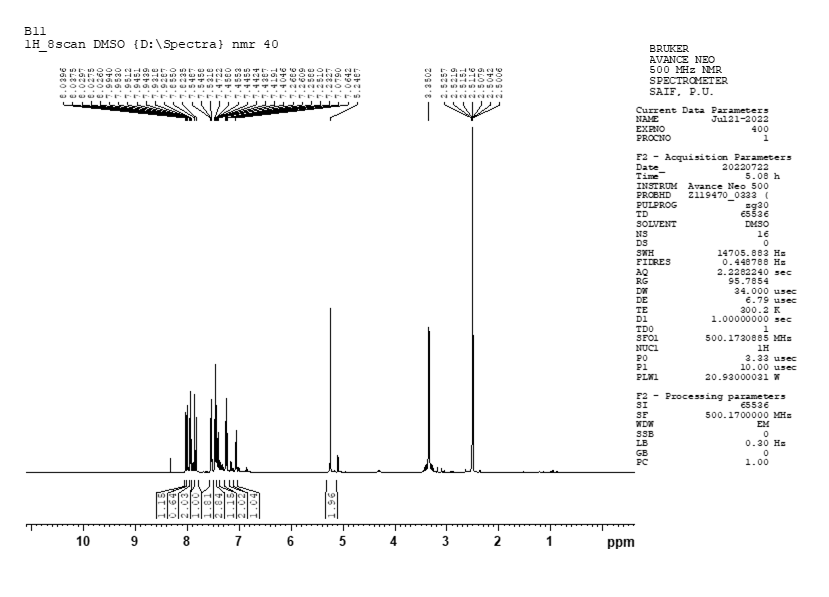


Figure S25 | ^1^H-NMR spectrum of compound **B11.**


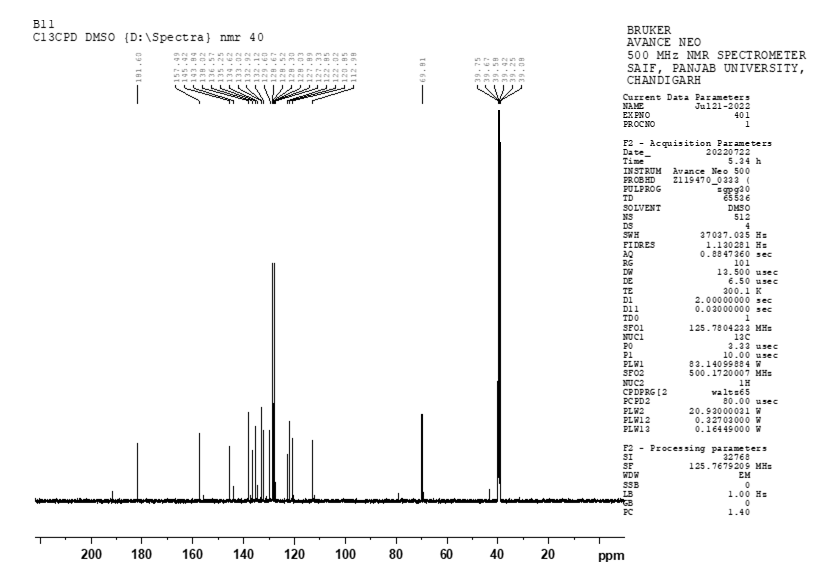


Figure S26 | ^13^C-NMR spectrum of compound **B11.**


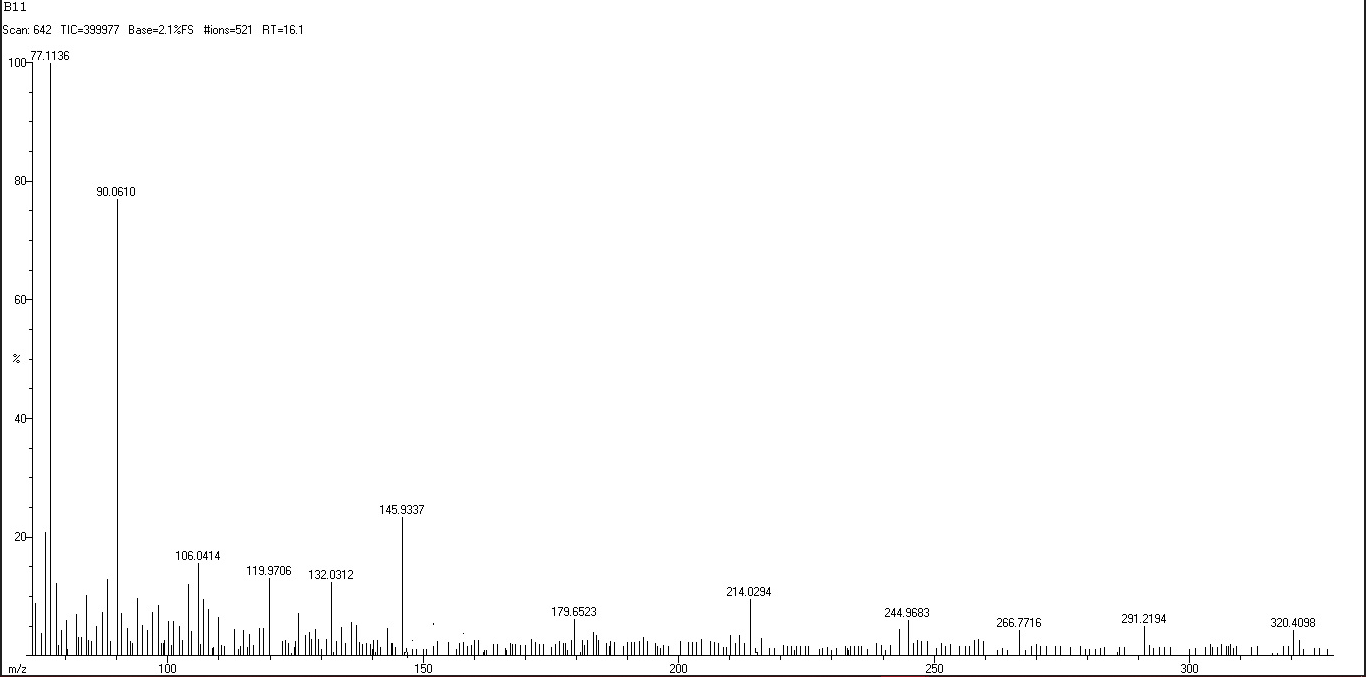


Figure S27 | HRMS of compound **B11.**

**(E)-3-(2-(benzyloxy)phenyl)-1-(4-ethoxyphenyl)prop-2-en-1-one [B14] :**^1^H NMR (500 MHz, DMSO) δ: 1.37 (3H,S,CH_3_-CH2-), 4.16-4.11 (2H, m, CH_3_-CH_2_-O-), 5.23 (2H,S,-O-CH_2_-C_6_H_5_-), 7.01-6.99 (2H, *J* = 10 MHz, Ar-H), 7.06-7.03 (1H, *J* = 15MHz, -CH_α_), 7.24-7.21 (2H, *J* = 10 MHz, Ar-H), 7.48-7.41 (4H, Ar-H), 7.55-7.54 (2H, *J* = 10 MHz, Ar-H), 7.99-7.88 (5H, Ar-H). ^13^C NMR (500 MHz, DMSO) δ: 181.60, 157.49, 143.84, 138.02, 136.57, 135.25, 132.2, 129.60, 128.67, 128.52, 128.30, 128.03, 127.89, 127.33, 122.85, 120.85, 112.98, 69.81.Molecular formula: C_24_H_22_O_3_ (HRMS), Calculated = 358.4296, Observed = 358.4399.


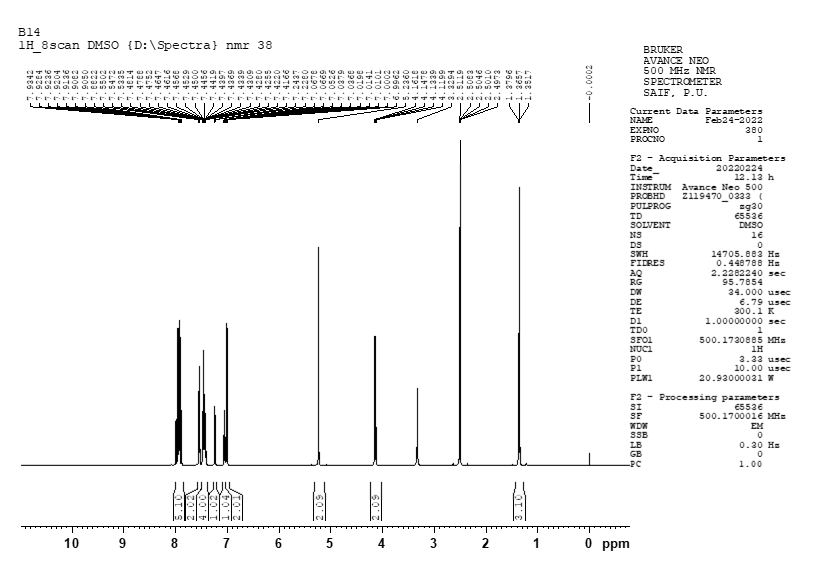


Figure S28 | ^1^H-NMR spectrum of compound **B14.**


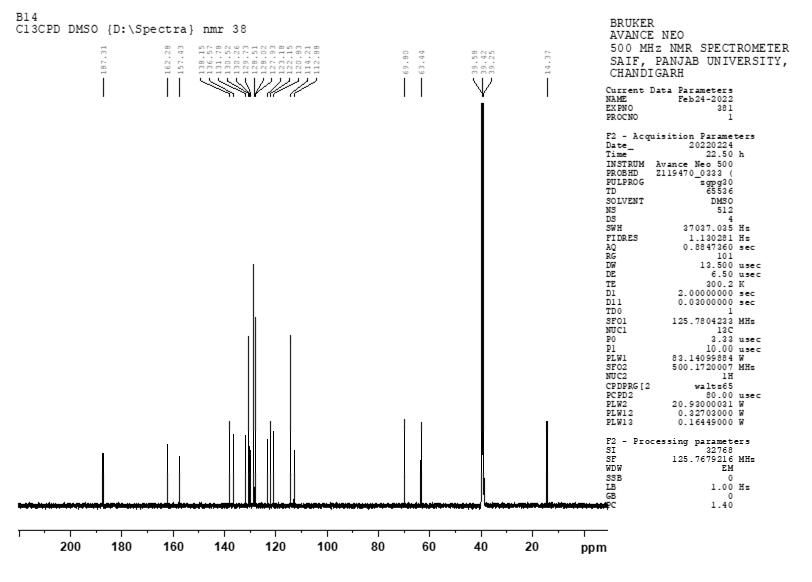


Figure S29 | ^13^C-NMR spectrum of compound **B14.**


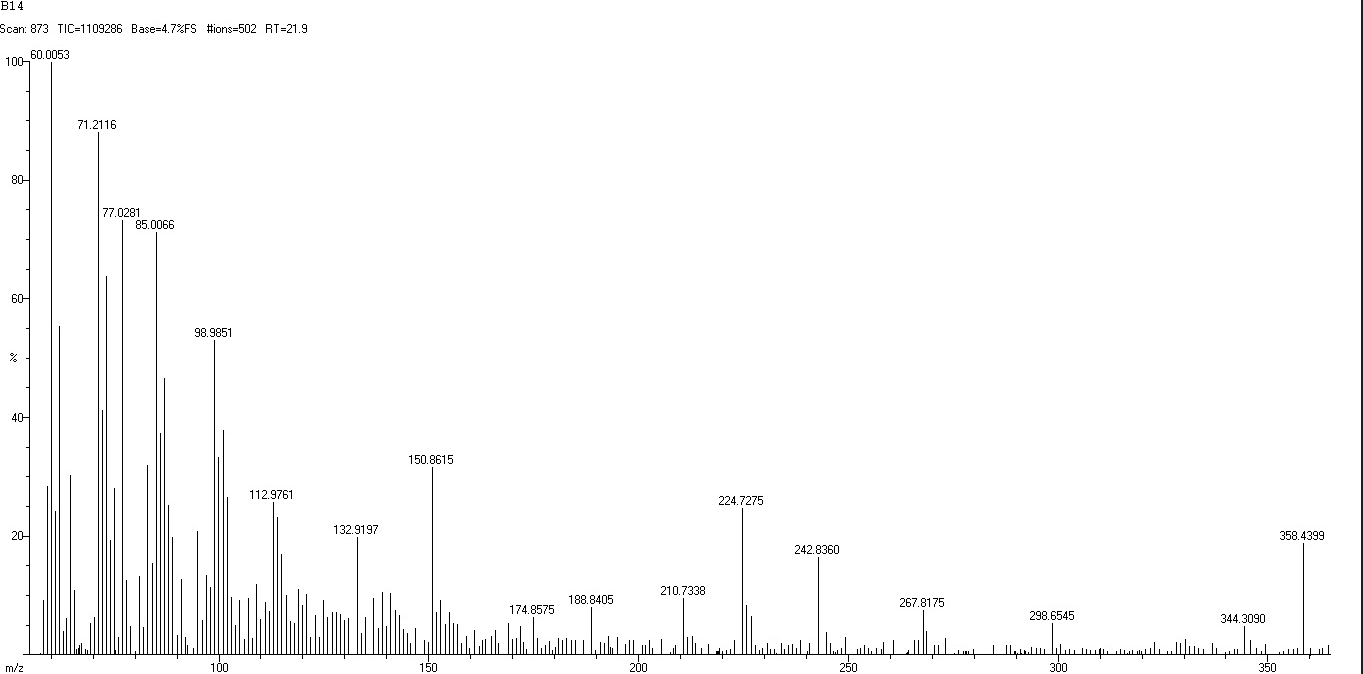


Figure S30 | HRMS of compound **B14.**

**(E)-3-(4-(benzyloxy)phenyl)-1-(4-ethoxyphenyl)prop-2-en-1-one [B15]****:** ^1^H NMR (500 MHz, DMSO) δ: 1.37-1.34 (3H,t, CH_3_-CH2-), 4.16-4.12 (2H, m, CH_3_-CH_2_-O-), 5.18 (2H,s,-O-CH_2_-C_6_H_5_-), 7.06-7.04 (2H, *J* = 10 MHz, Ar-H), 7.10-7.08 (2H, *J* = 10 MHz, Ar-H), 7.35-7.33(1H, *J* = 10 MHz, Ar-H), 7.47-7.39 (4H, Ar-H), 7.69-7.66 (1H, *J* = 15MHz, - CH_α_),7.82-7.79 (1H, *J* = 15MHz, -CH_β_), 7.84-7.83 (2H, Ar-H), 8.14-8.13 (2H, Ar-H). ^13^C NMR (500 MHz, DMSO) δ: 187.11, 162.27, 160.15, 142.88, 136.61, 130.68, 130.51, 130.41, 128.35, 127.82, 127.65, 127.56, 119.51, 115.09, 114.20, 79.06, 69.28, 63.43, 39.58, 39.42, 39.25, 14.39. Molecular formula: C_24_H_22_O_3_ (HRMS), Calculated = 358.4296, Observed = 358.4398


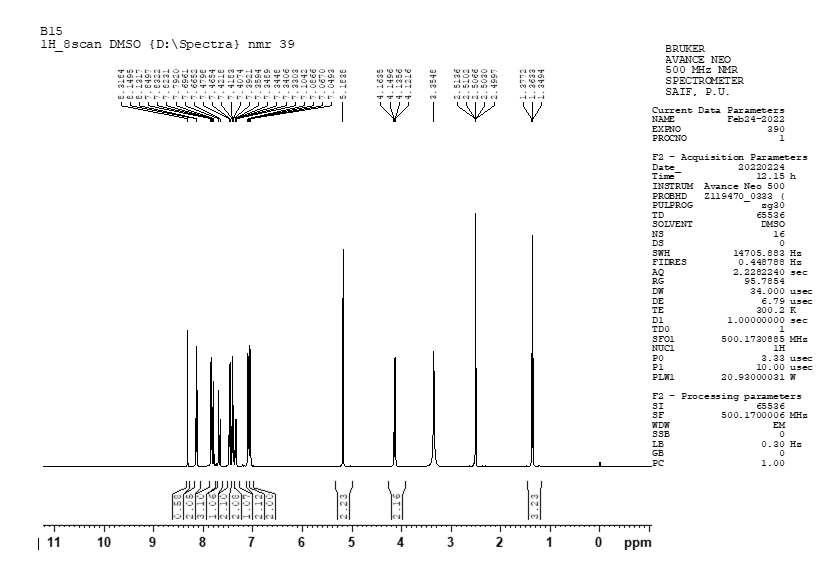


Figure S31 | ^1^H-NMR spectrum of compound **B15.**


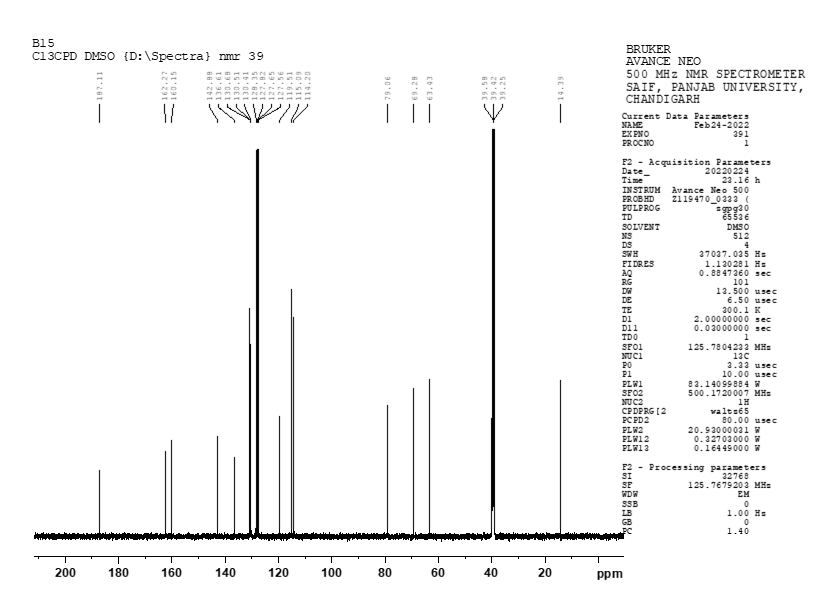


Figure S32 | ^13^C-NMR spectrum of compound **B15.**


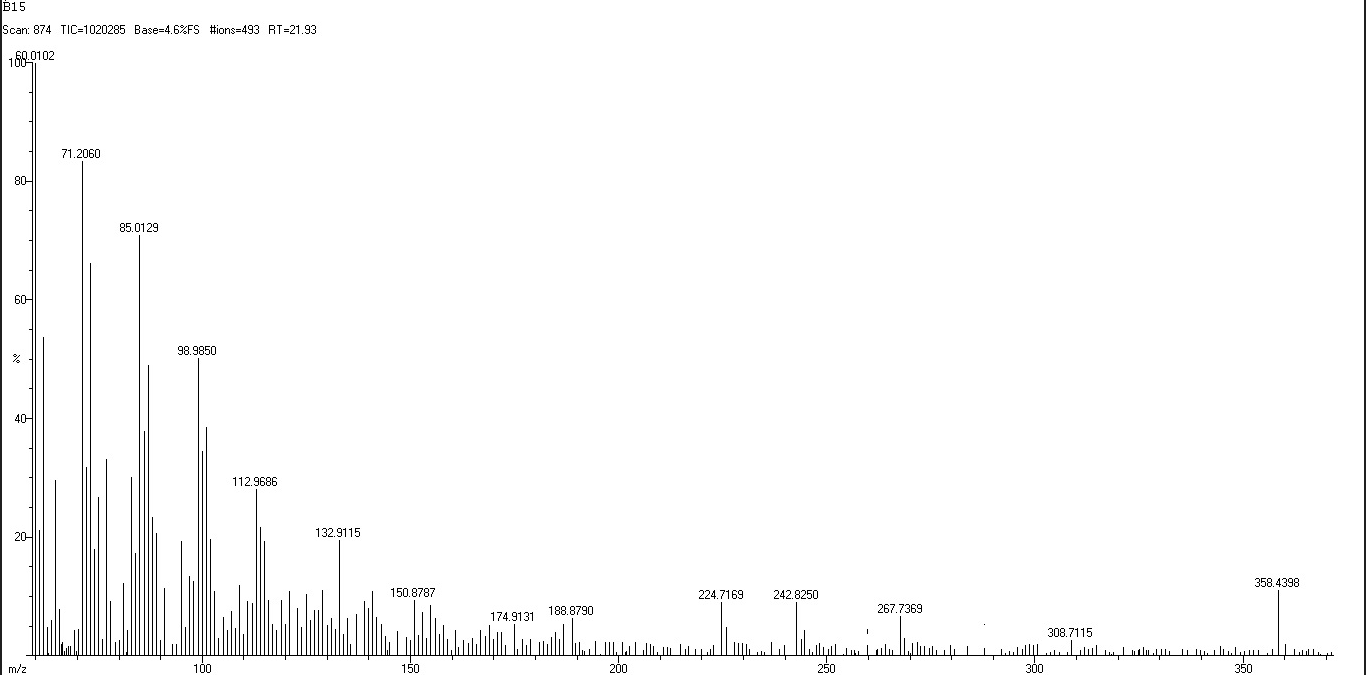


Figure S33 | HRMS of compound **B15.**

**Table S1**. MM-GBSA based binding free energy for **B10**-MAO-B complex

| **Time**  **(ns)** | **∆G_Bind_** | **∆G_Bind_Coul_** | **∆G_BindCov_** | **∆G_Bind_Hbond_** | **∆G_Bind_Lip_** | **∆G_bind_pack_** | **∆G_bindsol_GB_** | **∆G_bind_vdw_** |
| --- | --- | --- | --- | --- | --- | --- | --- | --- |
| 0 | -60.83 | -1.68 | 4.93 | 0.00 | -37.81 | -7.81 | 37.87 | -57.63 |
| 1 | -62.26 | -8.77 | 3.04 | -0.16 | -37.63 | -4.81 | 39.29 | -54.53 |
| 2 | -63.20 | -8.10 | 3.57 | -0.02 | -38.33 | -5.30 | 38.99 | -55.31 |
| 3 | -78.68 | -11.23 | 0.53 | -0.42 | -35.34 | -4.65 | 27.21 | -56.08 |
| 4 | -80.96 | -23.27 | 7.97 | -1.05 | -34.93 | -5.50 | 32.74 | -58.22 |
| 5 | -79.14 | -19.00 | 6.52 | -1.54 | -36.01 | -5.41 | 36.32 | -61.31 |
| 6 | -72.50 | -13.66 | 4.89 | -0.59 | -37.09 | -4.94 | 39.50 | -61.91 |
| 7 | -86.04 | -19.05 | 2.67 | -1.77 | -35.41 | -5.08 | 29.35 | -58.06 |
| 8 | -86.59 | -20.26 | 6.32 | -1.54 | -38.47 | -5.09 | 33.07 | -61.92 |
| 9 | -83.76 | -16.43 | 7.00 | -0.89 | -39.15 | -5.30 | 31.01 | -61.30 |
| 10 | -83.34 | -16.13 | 6.03 | -0.87 | -39.64 | -6.04 | 35.23 | -63.23 |
| 11 | -81.33 | -11.25 | 3.34 | -0.83 | -39.00 | -5.33 | 35.36 | -64.92 |
| 12 | -81.03 | -18.77 | 6.05 | -1.19 | -37.84 | -4.84 | 37.11 | -62.84 |
| 13 | -77.81 | -16.72 | 5.09 | -1.40 | -39.18 | -6.11 | 38.99 | -59.78 |
| 14 | -73.15 | -14.78 | 4.82 | -0.99 | -37.04 | -4.31 | 35.71 | -57.87 |
| 15 | -69.22 | -16.72 | 9.67 | -0.95 | -35.00 | -4.77 | 37.23 | -59.98 |
| 16 | -75.04 | -19.79 | 7.35 | -1.72 | -37.66 | -5.00 | 39.33 | -58.85 |
| 17 | -79.49 | -19.80 | 6.34 | -1.51 | -35.17 | -5.30 | 30.25 | -55.60 |
| 18 | -75.50 | -17.13 | 6.50 | -1.26 | -36.58 | -4.89 | 37.61 | -61.06 |
| 19 | -75.22 | -10.95 | 6.77 | -1.39 | -36.15 | -5.10 | 30.07 | -59.76 |
| 20 | -76.22 | -12.28 | 5.06 | -1.08 | -38.19 | -5.93 | 36.48 | -61.57 |
| 21 | -59.82 | -3.74 | 5.74 | -0.25 | -36.94 | -6.81 | 42.13 | -61.24 |
| 22 | -71.77 | -13.87 | 7.39 | -1.05 | -36.15 | -7.91 | 37.11 | -58.58 |
| 23 | -82.04 | -17.54 | 5.84 | -1.61 | -38.61 | -6.48 | 33.34 | -58.29 |
| 24 | -65.66 | -2.97 | 4.54 | -0.27 | -36.68 | -6.98 | 34.48 | -59.08 |
| 25 | -76.94 | -15.95 | 6.12 | -0.79 | -38.67 | -5.84 | 33.96 | -57.07 |
| 26 | -76.68 | -13.66 | 7.17 | -0.69 | -37.64 | -6.52 | 35.98 | -62.62 |
| 27 | -78.15 | -16.92 | 3.90 | -0.56 | -34.35 | -3.92 | 28.89 | -56.48 |
| 28 | -78.31 | -13.33 | -0.66 | -0.52 | -34.63 | -4.50 | 28.98 | -54.95 |
| 29 | -74.18 | -10.64 | 0.29 | -0.48 | -34.37 | -3.05 | 29.96 | -57.19 |
| 30 | -72.63 | -13.29 | 0.88 | -0.54 | -32.43 | -4.73 | 31.50 | -55.33 |
| 31 | -76.64 | -14.82 | 6.86 | -0.52 | -35.52 | -6.35 | 30.95 | -58.53 |
| 32 | -71.12 | -16.36 | 7.54 | -0.88 | -34.00 | -5.43 | 34.07 | -57.37 |
| 33 | -86.42 | -19.43 | 6.17 | -1.14 | -36.77 | -6.13 | 28.07 | -58.49 |
| 34 | -68.18 | -11.30 | 6.33 | -1.08 | -34.31 | -3.88 | 31.12 | -56.36 |
| 35 | -78.25 | -16.35 | 6.83 | -1.08 | -35.53 | -5.25 | 29.58 | -57.75 |
| 36 | -69.71 | -13.03 | 7.18 | -1.15 | -33.15 | -3.56 | 27.56 | -54.86 |
| 37 | -76.66 | -14.07 | 4.27 | -1.10 | -35.31 | -6.15 | 31.56 | -57.16 |
| 38 | -73.35 | -11.89 | 4.81 | -1.04 | -33.21 | -4.86 | 27.75 | -56.21 |
| 39 | -67.56 | -11.69 | 5.28 | -0.90 | -34.45 | -5.37 | 29.62 | -51.35 |
| 40 | -74.60 | -15.61 | 4.65 | -1.30 | -34.33 | -3.39 | 29.69 | -55.61 |
| 41 | -78.02 | -16.65 | 4.91 | -1.43 | -34.81 | -3.93 | 29.76 | -57.16 |
| 42 | -64.67 | -10.96 | 7.26 | -0.89 | -34.60 | -4.22 | 33.43 | -55.98 |
| 43 | -70.28 | -12.32 | 5.50 | -0.82 | -33.39 | -4.05 | 33.66 | -60.17 |
| 44 | -74.88 | -13.12 | 5.10 | -0.99 | -35.87 | -4.77 | 32.58 | -59.11 |
| 45 | -67.65 | -13.11 | 5.20 | -1.01 | -34.05 | -4.10 | 34.94 | -56.83 |
| 46 | -66.13 | -8.76 | 4.89 | -1.00 | -33.45 | -4.04 | 32.92 | -57.99 |
| 47 | -73.68 | -11.94 | 5.27 | -1.05 | -35.08 | -5.53 | 31.18 | -57.84 |
| 48 | -72.76 | -12.94 | 5.26 | -0.89 | -34.39 | -4.70 | 33.19 | -59.59 |
| 49 | -63.23 | -11.80 | 5.02 | -1.05 | -32.54 | -3.98 | 35.99 | -56.17 |
| 50 | -73.51 | -18.03 | 5.26 | -1.18 | -33.75 | -5.07 | 33.79 | -55.83 |
| 51 | -75.14 | -14.18 | 4.09 | -1.13 | -34.70 | -4.18 | 33.09 | -59.42 |
| 52 | -75.41 | -15.57 | 4.67 | -1.30 | -32.74 | -3.67 | 28.51 | -56.59 |
| 53 | -76.74 | -15.86 | 4.97 | -1.27 | -33.70 | -5.17 | 30.74 | -57.76 |
| 54 | -77.89 | -14.81 | 3.73 | -0.96 | -35.03 | -4.51 | 29.56 | -57.17 |
| 55 | -77.20 | -12.66 | 5.01 | -1.09 | -36.12 | -3.82 | 28.64 | -58.44 |
| 56 | -84.71 | -18.18 | 4.43 | -1.29 | -35.32 | -4.06 | 26.39 | -57.96 |
| 57 | -77.01 | -13.97 | 5.50 | -1.08 | -37.01 | -4.82 | 32.21 | -59.13 |
| 58 | -79.19 | -17.43 | 3.75 | -1.14 | -35.65 | -4.89 | 30.38 | -55.51 |
| 59 | -73.93 | -10.59 | 0.37 | -0.54 | -35.38 | -4.23 | 30.82 | -55.69 |
| 60 | -76.14 | -14.05 | 2.73 | -0.54 | -35.14 | -5.42 | 30.99 | -56.02 |
| 61 | -82.10 | -15.77 | 3.22 | -0.55 | -36.83 | -5.62 | 28.47 | -56.31 |
| 62 | -82.15 | -16.77 | 2.95 | -0.55 | -36.87 | -5.37 | 29.55 | -56.39 |
| 63 | -76.26 | -12.57 | 1.84 | -0.52 | -37.41 | -7.85 | 31.62 | -52.66 |
| 64 | -76.60 | -13.99 | 1.88 | -0.58 | -37.25 | -6.28 | 30.90 | -52.58 |
| 65 | -80.62 | -16.00 | 1.80 | -0.53 | -36.94 | -6.74 | 29.00 | -52.51 |
| 66 | -76.47 | -16.17 | 3.07 | -0.51 | -38.47 | -5.29 | 32.28 | -52.68 |
| 67 | -77.73 | -13.74 | 3.32 | -0.71 | -37.62 | -5.34 | 29.32 | -54.26 |
| 68 | -70.92 | -15.69 | 0.47 | -0.53 | -36.77 | -3.93 | 34.37 | -50.15 |
| 69 | -77.27 | -16.92 | 1.89 | -0.56 | -34.42 | -5.40 | 28.15 | -51.30 |
| 70 | -71.62 | -13.68 | 1.22 | -0.43 | -35.39 | -4.55 | 33.77 | -53.86 |
| 71 | -81.95 | -15.13 | 2.02 | -0.52 | -38.45 | -4.28 | 27.12 | -54.01 |
| 72 | -75.70 | -15.23 | 2.30 | -0.50 | -36.88 | -4.55 | 29.79 | -51.93 |
| 73 | -78.47 | -17.47 | 4.45 | -1.34 | -35.13 | -4.49 | 29.67 | -55.46 |
| 74 | -67.64 | -12.43 | 4.50 | -1.29 | -31.56 | -2.67 | 32.21 | -57.69 |
| 75 | -75.54 | -12.46 | 6.90 | -1.13 | -35.46 | -4.11 | 28.46 | -59.04 |
| 76 | -80.16 | -13.96 | 3.87 | -1.41 | -35.45 | -4.39 | 27.63 | -57.74 |
| 77 | -73.53 | -7.65 | 2.47 | -0.06 | -34.27 | -4.74 | 29.76 | -60.33 |
| 78 | -65.29 | -12.87 | 0.40 | -0.56 | -30.68 | -4.25 | 36.28 | -54.90 |
| 79 | -76.99 | -7.32 | 4.23 | -0.42 | -34.36 | -3.95 | 25.74 | -62.21 |
| 80 | -82.74 | -15.62 | -0.01 | -0.54 | -36.51 | -5.36 | 28.91 | -54.91 |
| 81 | -70.95 | -9.53 | 0.19 | -0.54 | -34.66 | -5.08 | 32.92 | -55.55 |
| 82 | -84.62 | -17.58 | 1.35 | -0.63 | -34.63 | -5.37 | 30.15 | -59.21 |
| 83 | -70.58 | -12.48 | -0.17 | -0.52 | -32.12 | -4.58 | 34.38 | -56.39 |
| 84 | -77.89 | -15.48 | 2.12 | -0.58 | -33.37 | -4.30 | 31.75 | -59.32 |
| 85 | -72.06 | -9.64 | 5.59 | -0.52 | -34.66 | -4.69 | 31.70 | -61.13 |
| 86 | -68.13 | -10.98 | 6.25 | -1.08 | -33.49 | -2.69 | 30.01 | -57.46 |
| 87 | -72.65 | -13.78 | 4.10 | -1.07 | -35.50 | -4.26 | 32.71 | -56.14 |
| 88 | -75.08 | -14.66 | 8.14 | -1.18 | -33.84 | -3.78 | 26.22 | -57.28 |
| 89 | -71.79 | -15.77 | 7.18 | -1.21 | -33.82 | -2.91 | 28.60 | -55.15 |
| 90 | -80.09 | -15.49 | 6.69 | -1.34 | -38.23 | -4.20 | 28.60 | -57.42 |
| 91 | -72.81 | -13.31 | 4.89 | -1.38 | -33.42 | -4.17 | 27.65 | -54.37 |
| 92 | -74.45 | -12.43 | 4.09 | -1.02 | -33.64 | -4.06 | 29.34 | -58.02 |
| 93 | -78.53 | -13.59 | 4.40 | -1.27 | -35.18 | -3.76 | 27.78 | -58.20 |
| 94 | -71.53 | -10.44 | 4.10 | -1.01 | -33.23 | -3.06 | 29.36 | -58.53 |
| 95 | -72.81 | -9.54 | 6.30 | -0.85 | -35.13 | -3.76 | 29.42 | -60.56 |
| 96 | -75.92 | -13.15 | 4.65 | -1.12 | -35.55 | -5.20 | 28.43 | -55.28 |
| 97 | -81.09 | -13.68 | 4.78 | -1.07 | -36.11 | -3.98 | 26.62 | -58.95 |
| 98 | -85.28 | -18.72 | 4.51 | -1.19 | -34.97 | -4.73 | 27.07 | -58.55 |
| 99 | -84.55 | -16.94 | 5.24 | -1.43 | -34.26 | -3.87 | 23.81 | -58.41 |
| 100 | -83.39 | -16.47 | 4.79 | -1.34 | -36.22 | -3.91 | 26.54 | -58.08 |
| **Max.** | -86.59 | -23.27 | -0.66 | -1.77 | -39.64 | -7.91 | 23.81 | -64.92 |
| **Min.** | -59.82 | -1.68 | 9.67 | 0.00 | -30.68 | -2.67 | 42.13 | -50.15 |
| **Avg.** | -75.37 | -14.00 | 4.46 | -0.92 | -35.55 | -4.84 | 31.62 | -57.44 |
| **Std.** | 5.85 | 3.58 | 2.15 | 0.39 | 1.84 | 1.04 | 3.65 | 2.78 |

**Table S2.** MM-GBSA based binding free energy for **B15**-MAO-B complex

| **Time**  **(ns)** | **∆G_Bind_** | **∆G_Bind_Coul_** | **∆G_BindCov_** | **∆G_Bind_Hbond_** | **∆G_Bind_Lip_** | **∆G_bind_pack_** | **∆G_bindsol_GB_** | **∆G_bind_vdw_** |
| --- | --- | --- | --- | --- | --- | --- | --- | --- |
| 0 | -73.97 | -5.96 | 6.26 | -0.27 | -41.99 | -6.49 | 38.85 | -65.61 |
| 1 | -81.49 | -10.56 | 1.59 | -0.75 | -41.03 | -4.91 | 36.89 | -63.95 |
| 2 | -76.49 | -12.00 | 2.45 | -0.33 | -41.74 | -5.01 | 42.13 | -63.21 |
| 3 | -75.52 | -9.35 | 1.66 | -0.53 | -41.71 | -5.12 | 43.56 | -65.27 |
| 4 | -85.82 | -13.60 | 5.54 | -0.66 | -43.33 | -5.42 | 37.05 | -66.61 |
| 5 | -87.75 | -10.62 | 5.51 | -0.55 | -44.25 | -5.70 | 34.53 | -67.89 |
| 6 | -88.49 | -9.84 | 5.19 | -0.57 | -42.82 | -4.86 | 29.83 | -66.64 |
| 7 | -78.94 | -4.85 | 4.93 | -0.59 | -41.42 | -4.84 | 31.57 | -64.96 |
| 8 | -79.45 | -12.29 | 5.42 | -0.64 | -40.55 | -3.95 | 35.20 | -63.85 |
| 9 | -79.24 | -7.73 | 5.13 | -0.25 | -43.77 | -6.14 | 39.59 | -67.29 |
| 10 | -80.17 | -9.45 | 5.08 | -0.39 | -42.20 | -5.33 | 35.26 | -64.37 |
| 11 | -88.17 | -11.22 | 4.93 | -0.51 | -43.09 | -5.46 | 31.96 | -66.00 |
| 12 | -84.30 | -7.30 | 5.13 | -0.47 | -42.67 | -5.58 | 31.28 | -65.91 |
| 13 | -78.25 | -9.32 | 5.90 | -0.63 | -39.87 | -4.84 | 30.13 | -60.79 |
| 14 | -91.66 | -13.45 | 4.16 | -0.86 | -43.13 | -5.38 | 31.51 | -65.69 |
| 15 | -95.26 | -13.46 | 3.23 | -0.67 | -42.34 | -5.51 | 27.91 | -65.59 |
| 16 | -80.72 | -13.13 | 6.17 | -0.61 | -39.59 | -4.62 | 31.82 | -61.93 |
| 17 | -79.49 | -11.62 | 6.12 | -0.54 | -39.59 | -5.11 | 33.12 | -63.04 |
| 18 | -86.21 | -16.24 | 4.14 | -0.55 | -44.99 | -7.98 | 41.59 | -63.36 |
| 19 | -70.82 | -12.04 | 2.75 | -0.52 | -34.94 | -6.12 | 33.57 | -54.70 |
| 20 | -84.16 | -15.04 | 5.42 | -0.76 | -43.82 | -7.70 | 43.30 | -66.73 |
| 21 | -87.01 | -12.83 | 3.93 | -0.68 | -44.79 | -7.21 | 38.92 | -65.52 |
| 22 | -88.51 | -14.15 | 3.35 | -0.67 | -44.15 | -7.63 | 37.71 | -64.14 |
| 23 | -87.28 | -14.17 | 3.77 | -0.83 | -43.14 | -7.26 | 33.74 | -60.55 |
| 24 | -88.31 | -17.00 | 7.74 | -0.60 | -44.43 | -7.28 | 34.30 | -62.22 |
| 25 | -92.14 | -13.03 | 3.27 | -0.60 | -46.92 | -8.00 | 36.90 | -64.91 |
| 26 | -97.50 | -17.90 | 2.97 | -0.69 | -46.15 | -7.33 | 36.10 | -65.67 |
| 27 | -86.98 | -13.10 | 2.47 | -0.58 | -42.69 | -7.46 | 36.95 | -63.72 |
| 28 | -93.56 | -11.95 | 2.05 | -0.54 | -46.34 | -7.31 | 33.91 | -64.55 |
| 29 | -86.85 | -13.13 | 2.16 | -0.65 | -44.40 | -7.27 | 39.51 | -64.25 |
| 30 | -88.94 | -13.35 | 2.78 | -0.55 | -44.66 | -7.50 | 35.71 | -62.54 |
| 31 | -87.88 | -8.55 | 1.43 | -0.59 | -45.95 | -6.86 | 38.56 | -67.08 |
| 32 | -87.90 | -13.86 | 4.21 | -0.57 | -45.30 | -7.37 | 37.35 | -63.53 |
| 33 | -82.06 | -8.89 | 1.50 | -0.58 | -43.29 | -6.74 | 39.53 | -64.75 |
| 34 | -85.82 | -13.80 | 2.48 | -0.67 | -42.98 | -7.04 | 40.61 | -65.60 |
| 35 | -91.69 | -9.99 | -0.81 | -0.57 | -47.15 | -7.66 | 38.15 | -64.84 |
| 36 | -90.14 | -12.59 | 0.28 | -0.56 | -45.41 | -7.54 | 37.63 | -63.12 |
| 37 | -88.49 | -13.64 | 0.34 | -0.56 | -45.80 | -7.53 | 40.70 | -63.19 |
| 38 | -86.12 | -10.34 | 0.52 | -0.54 | -44.69 | -6.48 | 39.15 | -64.92 |
| 39 | -94.12 | -11.07 | 0.97 | -0.37 | -46.40 | -8.36 | 41.56 | -71.62 |
| 40 | -101.88 | -14.74 | 0.17 | -0.60 | -45.77 | -8.10 | 34.67 | -68.67 |
| 41 | -92.94 | -13.88 | 2.74 | -0.57 | -46.01 | -8.26 | 41.13 | -69.26 |
| 42 | -92.52 | -9.42 | -1.26 | -0.58 | -45.70 | -7.27 | 37.96 | -67.42 |
| 43 | -94.38 | -10.55 | 3.70 | -0.55 | -47.26 | -7.83 | 36.09 | -69.16 |
| 44 | -91.55 | -13.44 | 2.27 | -0.47 | -45.22 | -8.13 | 39.72 | -67.44 |
| 45 | -95.38 | -11.83 | 2.00 | -0.56 | -48.11 | -8.07 | 36.41 | -66.41 |
| 46 | -91.41 | -12.17 | 1.86 | -0.56 | -45.23 | -7.91 | 39.13 | -67.71 |
| 47 | -86.59 | -10.86 | 0.89 | -0.59 | -45.57 | -7.64 | 42.54 | -66.53 |
| 48 | -101.42 | -14.82 | 0.75 | -0.57 | -48.90 | -7.99 | 38.30 | -69.35 |
| 49 | -98.13 | -14.78 | 0.44 | -0.57 | -46.76 | -8.33 | 38.35 | -67.65 |
| 50 | -92.64 | -13.92 | 1.38 | -0.54 | -45.62 | -7.86 | 38.99 | -66.24 |
| 51 | -95.61 | -10.73 | 0.16 | -0.54 | -44.57 | -8.27 | 34.97 | -67.79 |
| 52 | -88.21 | -12.77 | 2.76 | -0.56 | -45.03 | -7.94 | 41.34 | -67.19 |
| 53 | -89.97 | -10.20 | 1.78 | -0.55 | -45.75 | -7.57 | 37.34 | -66.19 |
| 54 | -84.46 | -8.54 | 2.60 | -0.54 | -46.57 | -8.00 | 41.75 | -66.33 |
| 55 | -93.20 | -9.92 | 1.40 | -0.63 | -47.06 | -6.89 | 34.85 | -66.12 |
| 56 | -80.83 | -9.17 | 3.01 | -0.54 | -46.48 | -7.42 | 40.42 | -61.82 |
| 57 | -83.85 | -8.38 | 1.04 | -0.56 | -43.63 | -7.62 | 37.38 | -63.24 |
| 58 | -87.82 | -14.46 | 2.19 | -0.55 | -44.75 | -7.23 | 38.73 | -62.93 |
| 59 | -85.76 | -11.81 | 1.99 | -0.51 | -45.98 | -7.81 | 42.28 | -65.09 |
| 60 | -92.75 | -15.26 | 3.70 | -0.54 | -45.00 | -8.11 | 40.68 | -69.40 |
| 61 | -79.13 | -9.90 | 3.59 | -0.43 | -44.91 | -7.82 | 43.52 | -64.36 |
| 62 | -84.51 | -11.15 | -0.36 | -0.55 | -41.70 | -8.03 | 37.96 | -61.86 |
| 63 | -93.05 | -14.63 | 1.58 | -0.56 | -44.56 | -8.06 | 38.22 | -66.21 |
| 64 | -92.39 | -13.28 | 2.89 | -0.59 | -45.16 | -7.37 | 35.59 | -65.63 |
| 65 | -89.65 | -10.38 | 1.41 | -0.49 | -46.58 | -7.17 | 38.50 | -66.12 |
| 66 | -94.00 | -9.35 | 1.40 | -0.56 | -45.60 | -7.84 | 32.08 | -65.30 |
| 67 | -91.51 | -13.08 | 1.85 | -0.57 | -46.57 | -8.10 | 38.35 | -64.57 |
| 68 | -93.68 | -10.86 | 4.47 | -0.60 | -44.82 | -7.54 | 33.73 | -69.24 |
| 69 | -91.27 | -13.21 | 2.18 | -0.61 | -44.81 | -7.60 | 37.59 | -65.97 |
| 70 | -88.98 | -11.53 | 3.56 | -0.52 | -47.01 | -8.86 | 41.80 | -67.60 |
| 71 | -90.28 | -11.37 | 2.67 | -0.54 | -46.81 | -8.04 | 41.68 | -69.04 |
| 72 | -85.87 | -5.23 | -0.29 | -0.52 | -45.86 | -8.15 | 38.82 | -65.82 |
| 73 | -84.55 | -7.75 | 2.05 | -0.54 | -44.69 | -7.22 | 39.41 | -66.99 |
| 74 | -84.11 | -8.43 | 3.34 | -0.52 | -43.92 | -7.78 | 37.91 | -65.87 |
| 75 | -91.76 | -11.29 | 3.48 | -0.56 | -46.78 | -7.64 | 36.74 | -66.88 |
| 76 | -84.79 | -12.36 | 0.80 | -0.56 | -42.85 | -6.79 | 42.70 | -66.89 |
| 77 | -88.68 | -15.67 | 1.59 | -0.56 | -45.84 | -7.95 | 44.31 | -65.73 |
| 78 | -82.93 | -13.43 | 1.55 | -0.57 | -43.57 | -6.91 | 43.44 | -64.61 |
| 79 | -85.81 | -8.50 | 2.82 | -0.20 | -45.85 | -7.69 | 41.14 | -68.71 |
| 80 | -87.93 | -9.11 | 0.69 | -0.55 | -45.21 | -8.31 | 35.64 | -62.25 |
| 81 | -89.89 | -9.34 | 3.30 | -0.51 | -46.13 | -7.50 | 37.37 | -68.25 |
| 82 | -90.62 | -12.18 | 1.52 | -0.61 | -44.81 | -7.90 | 39.75 | -67.56 |
| 83 | -91.24 | -13.16 | 0.79 | -0.54 | -45.19 | -8.14 | 37.85 | -64.02 |
| 84 | -84.51 | -7.93 | -0.19 | -0.58 | -43.92 | -8.35 | 40.76 | -65.48 |
| 85 | -92.00 | -12.25 | 2.83 | -0.57 | -45.86 | -8.73 | 40.42 | -69.02 |
| 86 | -95.46 | -11.38 | 2.98 | -0.56 | -46.48 | -7.84 | 36.34 | -69.68 |
| 87 | -81.88 | -6.61 | 1.19 | -0.57 | -43.01 | -8.18 | 39.27 | -65.14 |
| 88 | -88.75 | -11.15 | 4.96 | -0.48 | -47.35 | -8.15 | 43.32 | -71.06 |
| 89 | -81.76 | -7.33 | -0.44 | -0.47 | -44.01 | -8.10 | 45.52 | -68.10 |
| 90 | -88.52 | -13.57 | 3.50 | -0.59 | -44.40 | -7.31 | 39.90 | -67.21 |
| 91 | -85.52 | -9.90 | 3.04 | -0.51 | -47.46 | -8.53 | 46.26 | -69.60 |
| 92 | -90.70 | -12.55 | 2.97 | -0.57 | -45.85 | -8.55 | 42.19 | -69.50 |
| 93 | -94.43 | -8.30 | 1.97 | -0.56 | -46.26 | -7.77 | 34.99 | -69.66 |
| 94 | -89.37 | -12.06 | -1.01 | -0.61 | -41.89 | -6.87 | 36.97 | -65.06 |
| 95 | -86.05 | -10.81 | 2.68 | -0.53 | -45.67 | -7.69 | 41.90 | -67.11 |
| 96 | -91.43 | -10.67 | 3.56 | -0.56 | -46.94 | -7.69 | 39.90 | -70.20 |
| 97 | -80.86 | -7.61 | 2.41 | -0.48 | -44.66 | -7.58 | 41.89 | -66.01 |
| 98 | -90.36 | -7.46 | 0.21 | -0.51 | -45.89 | -7.40 | 34.67 | -65.16 |
| 99 | -92.16 | -7.97 | 2.20 | -0.38 | -46.88 | -7.53 | 37.85 | -70.63 |
| 100 | -95.22 | -10.32 | 4.01 | -0.56 | -47.76 | -7.56 | 35.77 | -70.00 |
| **Max.** | -101.88 | -17.90 | -1.26 | -0.86 | -48.90 | -8.86 | 27.91 | -71.62 |
| **Min.** | -70.82 | -4.85 | 7.74 | -0.20 | -34.94 | -3.95 | 46.26 | -54.70 |
| **Avg.** | -87.97 | -11.36 | 2.59 | -0.55 | -44.67 | -7.25 | 38.05 | -65.94 |
| **Std.** | 5.70 | 2.59 | 1.82 | 0.10 | 2.15 | 1.07 | 3.62 | 2.61 |

**Table S3.** Correlation matrix with all QSARINS models with 1 to 5 variables, along with included descriptor, training, and prediction.


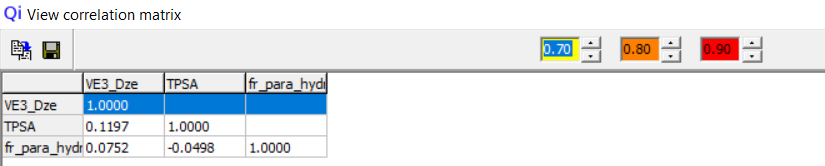


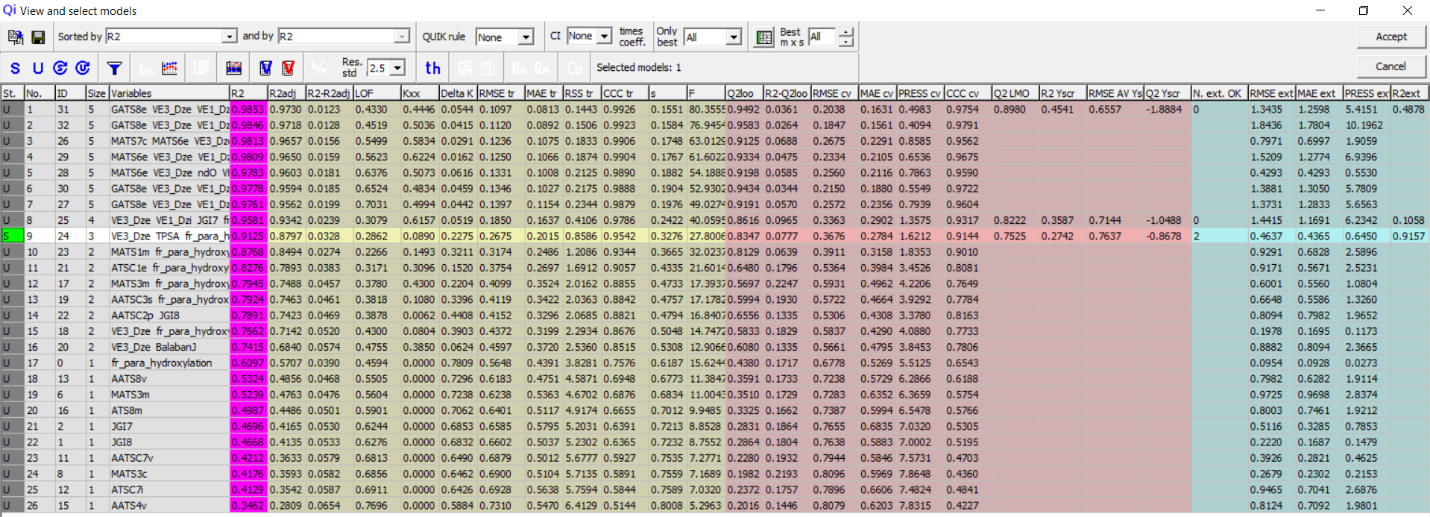


Training: 12 Prediction: 3 Variables: 382

Excluded: 0 Missing: 0 Unknown: 0
